# *Mentha haplocalyx* Briq. (Mint): a comprehensive review on the botany, traditional uses, nutritional value, phytochemistry, health benefits, and applications

**DOI:** 10.1186/s13020-024-01037-2

**Published:** 2024-12-12

**Authors:** Hai-Peng Tang, En-Lin Zhu, Qian-Xiang Bai, Shuang Wang, Zhi-Bin Wang, Meng Wang, Hai-Xue Kuang

**Affiliations:** 1 Key Laboratory of Basic and Application Research of Beiyao, Heilongjiang University of Chinese Medicine, Harbin, 150000 China; 2 Clinical Medical College of Acupuncture Moxibustion and Rehabilitation, Guangzhou University of Chinese Medicine, Guangzhou, 510006 China

**Keywords:** *Mentha haplocalyx* Briq., Traditional uses, Nutritional value, Phytochemistry, Health benefits, Applications

## Abstract

*Mentha haplocalyx* Briq. (*M. haplocalyx*), a notable member of the Lamiaceae family, occupies a significant role in the realm of health foods and botanical medicines. Traditionally, it has been employed to address various diseases, including colds, coughs, fever, indigestion, asthma, and influenza. Recent phytochemical investigations have identified the presence of terpenoids, flavonoids, phenolic acids, anthraquinones, alkanes, and polysaccharides in *M. haplocalyx*, with terpenoids being the primary bioactive constituents. Notably, both in vitro and in vivo studies have demonstrated its diverse health benefits, such as neuroprotective, anti-asthmatic, anti-inflammatory, gut health improvement, hypoglycemic, anti-aging, anti-bacterial, and antioxidant effects. Additionally, *M. haplocalyx* is a rich source of carbohydrates, dietary fiber, amino acids, minerals, and vitamins, further underscoring its nutritional value. A thorough literature review was conducted using databases like PubMed, Google Scholar, Web of Science, and China National Knowledge Infrastructure (CNKI) to consolidate existing knowledge on *M. haplocalyx*. This review synthesizes recent advancements in the botany, traditional uses, nutritional value, phytochemistry, health benefits, and research on the edible uses of *M. haplocalyx*. Furthermore, the commercial potential and future research opportunities for *M. haplocalyx* are briefly explored, with the goal of fostering continued interest in this multifunctional plant and inspiring future research and commercial endeavors.

**Graphical Abstract:**

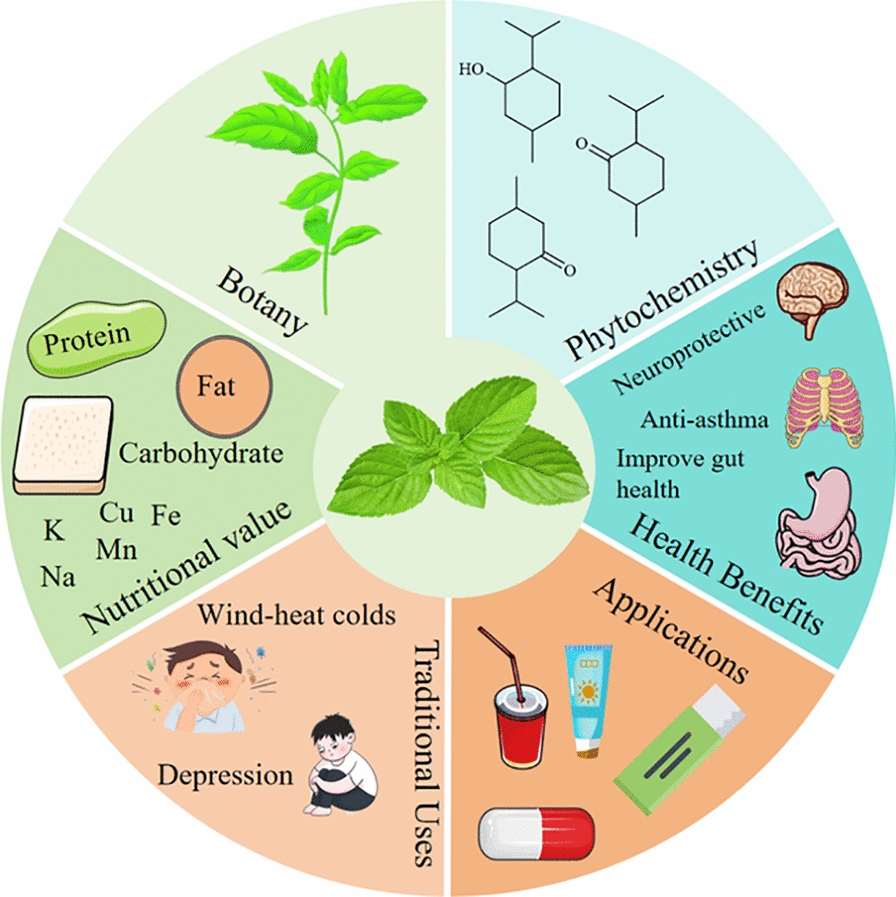

## Introduction

*Mentha haplocalyx* Briq. (*M. haplocalyx*), a notable aromatic herb within the Lamiaceae family, is highly valued for its edible and medicinal properties and is cultivated extensively across the globe [1, 2]. Predominantly found in subtropical and temperate regions of the Northern Hemisphere, it thrives especially in the humid areas of China, Korea, and Japan [3, 4]. In traditional medicine, the aerial parts of *M. haplocalyx*, particularly the stems and leaves, have been employed as herbal remedies for wounds, swollen glands, colds, coughs, fevers, indigestion, asthma, and influenza [5, 6]. Moreover, it is recognized in the 2020 edition of the Pharmacopoeia of the People’s Republic of China (Ch.P 2020), underscoring its importance in traditional medicinal practices and its integration into various pharmaceutical products [7]. *M. haplocalyx* is not only renowned for its therapeutic properties but also as a flavorful ingredient with health-promoting benefits [8]. Its leaves are widely used as flavoring agents in chewing gum, candies, beverages, and tobacco, imparting a refreshing aroma and taste. Additionally, *M. haplocalyx* is a key ingredient in oral hygiene products, cosmetics, herbal teas, and health beverages, attributed to its recognized health benefits [9–11]. Economically, *M. haplocalyx* is regarded as a valuable crop with significant potential for commercial exploitation, serving as an essential component in agricultural product development [12]. Cultivation has thus become the primary method for meeting the growing demand for *M. haplocalyx*. With over 2,000 years of cultivation history in ancient China, it has long been considered a significant spice and medicinal crop [13]. Contemporary pharmaceutical research largely concentrates on *M. haplocalyx*'s applications in neurological and respiratory disorders [14, 15], highlighting its contributions to both traditional medicine and modern drug development [16]. Beyond its medicinal attributes, *M. haplocalyx* offers considerable nutritional benefits, being a rich source of dietary fiber, vitamins, trace minerals, carbohydrates, and other essential nutrients. These qualities have attracted the attention of both nutrition researchers and health-conscious consumers [17, 18].

Throughout its long history of use, *M. haplocalyx* has been developed as a crucial source of functional foods and bioactive ingredients in traditional Chinese medicine (TCM) [19]. It remains a popular choice among consumers, both as a vegetable and a functional food, due to its refreshing flavor and nutritional richness [8, 20]. Notably, the essential oil derived from *M. haplocalyx* is a multifunctional substance with extensive medicinal and health benefits [21–24]. Historically, this essential oil has been esteemed for its efficacy in treating central nervous system disorders, leading to a focus on its volatile components in scientific studies [25]. However, recent findings suggest that non-volatile constituents, such as polyphenols and flavonoids, also play a vital role in clinical treatments, particularly for respiratory, reproductive, and digestive system disorders [26]. In summary, *M. haplocalyx* offers substantial benefits to human health, owing to its rich nutritional profile and therapeutic properties.

Historically, *M. haplocalyx* has been associated with numerous health benefits, including neuroprotection, anti-asthmatic effects, gut health improvement, hypoglycemic activity, anti-inflammatory properties, anti-aging effects, anti-bacterial, and antioxidant capabilities [4]. The phytochemical composition of medicinal plants forms the basis for disease prevention and treatment, driving scientists to discover new and more effective therapeutic agents [27–29]. A significant number of phytochemical compounds have been isolated and identified from *M. haplocalyx*, with terpenoids and other volatile components recognized as their primary bioactive constituents. In addition to terpenoids, *M. haplocalyx* contains phenols, flavonoids, anthraquinones, polysaccharides, alkanes, and various other phytochemicals [2, 9]. As research advances, there is an increasing focus on the chemical analysis and biological activities of *M. haplocalyx*. The identification of diverse phytochemicals and the exploration of their pharmacological activities lay a robust foundation for their further exploitation and utilization.

In recent years, research on *M. haplocalyx* has expanded across multiple disciplines. Despite this, no existing review article comprehensively addresses all facets of this plant. A thorough review of its research progress is essential for optimizing the utilization of this medicinal resource. Thus, this paper aims to present a comprehensive overview of *M. haplocalyx* research conducted over the past two decades, encompassing aspects such as botany, traditional uses, nutritional value, phytochemistry, health benefits, and potential applications. It is hoped that this paper will provide researchers with a broad understanding of *M. haplocalyx*'s research trajectory and serve as a valuable reference for future studies and applications.

## Botany

The *Mentha* genus, known for its rich species diversity and wide distribution, thrives predominantly in temperate and subtropical regions worldwide. Among its species, several are renowned for their medicinal and culinary value, including *M. haplocalyx*, *M. piperita*, *M. arvensis*, *M. longifolia*, *M. spicata*, and *M. aquatica*. *M. piperita*, typically growing to a height of 30–100 cm, is characterized by smooth stems and leaves measuring 4–9 cm in length and 1.5–4 cm in width. Its flowers, purple and arranged in whorled clusters, distinguish it from *M. haplocalyx*, which has rougher stems, smaller leaves, and lighter-colored flowers. *M. arvensis*, on the other hand, generally reaches a height of 10–60 cm, with paired leaves 2–6 cm long and 1–2 cm wide, and pale purple or pink flowers. *M. longifolia* can attain heights of 40–120 cm, with long, elliptical leaves measuring 5–10 cm in length and 1.5–3 cm in width, and pale purple or white flowers that grow in dense clusters. *M. spicata* usually grows to 30–100 cm, with leaves 5–9 cm long and 1.5–3 cm wide, and its flowers, white or pink, are borne on slender spikes. *M. aquatica*, a perennial herb with rhizomes, can grow up to 90 cm tall, featuring green or purple square stems, a fibrous root system, and small pink or purple flowers [30]. Despite their widespread use across various regions, these *Mentha* species exhibit significant differences in phytochemical composition and bioactive properties compared to *M. haplocalyx*. Species of the *Mentha* genus commonly contain a variety of natural compounds, including terpenoids, flavonoids, and phenolic acids. Specifically, the chemical composition of *M. piperita* and *M. spicata* also includes lignans. Additionally, *M. longifolia* contains cinnamates and ceramides in its chemical profile. These differences in chemical composition contribute to the distinct biological activities of each species. As one of the earliest introduced and cultivated plants in China, *M. haplocalyx* enjoys broad distribution and a long-standing history of medicinal use, earning its inclusion in the Ch.P 2020. Notably, due to the stringent requirements for clinical safety, efficacy, and quality control, *M. haplocalyx* remains the only species from the *Mentha* genus included in Ch.P 2020 to date.

**Fig. 1:**
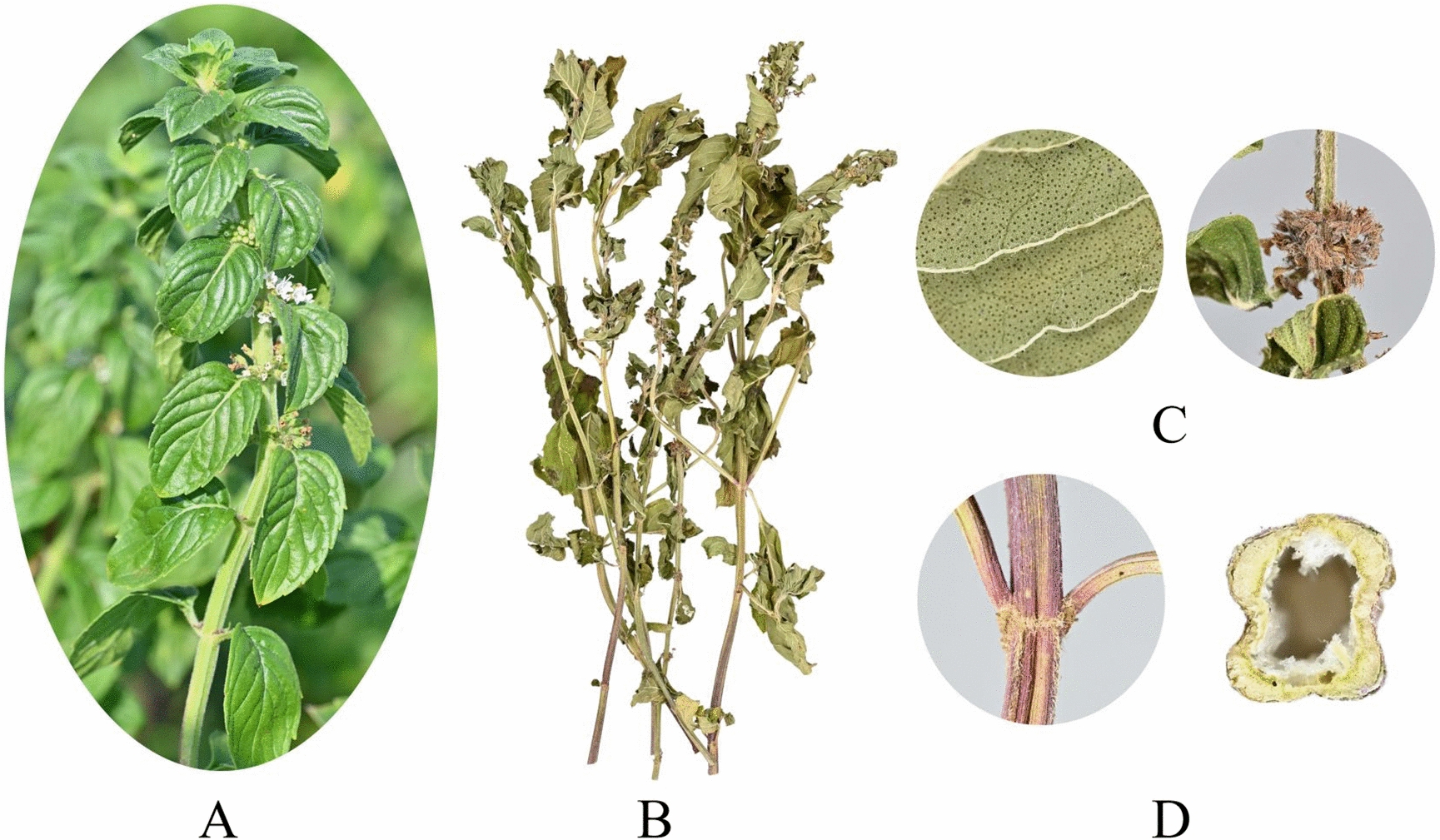
Plant morphology of *M. haplocalyx*. **A** Whole plants, **B** Dry medicinal parts, **C** Flowers, **D** Stems

Ch.P 2020 includes *M. haplocalyx*, specifically its dried aerial parts. *M. haplocalyx* is a perennial medicinal herb that thrives in humid environments, often found in wetlands near water, and can grow at altitudes up to 3,500 m [31]. According to online records from China’s flora (http://www.cn-flora.ac.cn/index.html, accessed on 25 May 2024), *M. haplocalyx* features erect stems that reach heights of 30–60 cm, with multiple nodes at the lower part, slender fibrous roots, and horizontally spreading rhizomes. The stems are sharply quadrangular, bearing four grooves, and are covered with inversely pubescent hairs on the upper part, while the lower part is pubescent only along the edges, branching extensively. The leaf blades are oblong-lanceolate, lanceolate, elliptic, or ovate-lanceolate, varying in shape, and measure 3–5 cm in length and 0.8–3 cm in width. They have acute apices, cuneate to subrounded bases, and sparsely coarse dentate margins above the base. The lateral veins number around 5–6 pairs, with a midrib that is slightly concave above and marked below, green on the upper surface. The leaves are sparsely pilose or nearly glabrous except along the veins, which are densely pilose, with petioles 2–10 mm long, ventrally concave, and puberulent. The axillary cymes are globose, approximately 18 mm in diameter, either pedicellate or sessile, with slender pedicels up to 3 mm long, puberulent or nearly glabrous. The calyx is tubular and bell-shaped, approximately 2.5 mm long, with a puberulent and glandular outer surface and a glabrous inner surface. It contains 10 inconspicuous veins and has 5 narrowly triangular subulate calyx teeth with long acute apices, each about 1 mm long. The corolla is lavender, 4 mm long, with a slightly puberulent outer surface and a puberulent inner surface below the throat, featuring a 4-lobed coronal eave. The upper lobe is 2-lobed and larger, while the remaining 3 lobes are subequal, oblong, with obtuse apices. The plant has four stamens, with the anterior pair extending beyond the corolla, approximately 5 mm long, with filamentous and glabrous filaments. The anthers are oval with parallel compartments, and the style slightly exceeds the stamens, with a nearly equal 2-lobed apex and subulate lobes. The flowering period occurs from July to September, with fruiting in October. The plant’s features are illustrated in Fig. 1 (https://ppbc.iplant.cn/). *M. haplocalyx* is typically harvested during the peak growth of stems and leaves or when flowers are fully bloomed, usually in summer and autumn. Harvesting on sunny days yields a higher volatile oil content, whereas harvesting on rainy days results in minimal content. After harvesting, *M. haplocalyx* must be promptly washed to remove surface impurities, soil, and pesticide residues, followed by drying in a well-ventilated, cool area until it reaches a semi-dry or dry state, reducing moisture content to prevent mold growth [32]. Post-drying, *M. haplocalyx* is cut into appropriately sized pieces or slices for further processing or use. It is crucial that harvesting and processing strictly follow operational procedures to ensure the quality and safety of *M. haplocalyx* products, meeting both culinary and medicinal standards.

## Traditional uses

*M. haplocalyx*, a medicinal and edible plant, has been traditionally used in China for over 2,000 years, dating back to the Han Dynasty. It was first documented in *Shen Nong's Materia Medica* as a treatment for symptoms such as wind-heat colds, headaches, and coughs [22]. Since ancient times, the exploration and development of TCM have been ongoing, strengthening its application in disease treatment and prevention, thereby bolstering confidence in the progress and innovation of TCM [33, 34]. *M. haplocalyx* was first included in the Ch.P 1963 and has long been recognized in folk culture as a commonly used TCM. According to TCM theory, *M. haplocalyx* is described as cold in nature, pungent in flavor, and associated with the lung and liver meridians. Its action on these meridians allows it to disperse wind-heat, clear the head, benefit the throat, promote rash eruption, and soothe liver qi. These properties, linked to its flavor and meridian affiliation, are critical in guiding the clinical application of herbal medicine within the TCM framework [35].

In clinical practice, *M. haplocalyx* is often combined with other herbs to enhance therapeutic effects, with the composition adjusted according to the symptoms being treated. Numerous prescriptions containing *M. haplocalyx* are currently used in TCM for treating conditions such as depression and atopic dermatitis. For example, Xiaoyaosan (XYS), a renowned classic TCM prescription for depressive disorders, includes *M. haplocalyx* as a key ingredient. TCM theory interprets depression as "liver qi stagnation," and XYS addresses this by targeting the underlying condition. Studies have demonstrated that *M. haplocalyx* has a significant effect on liver damage [36], further supporting its role in regulating liver qi and contributing to depression treatment within the XYS formulation [37–39]. Additionally, *M. haplocalyx* is a key component in a tri-herb formula, used topically in a 1:1:1 ratio with *Paeonia suffruticosa* Andr. and *Calendula officinalis* L. for treating atopic dermatitis. Research has confirmed that *M. haplocalyx* significantly reduces skin irritation in this formulation, making it a promising option for managing specific dermatitis conditions [40]. Beyond China, *M. haplocalyx* is also a significant element in ethnic medicine in countries such as Japan and Korea, where it is used to treat indigestion and respiratory infections, as noted in the Japanese and Korean Pharmacopoeias [3]. In Europe and America, *M. haplocalyx* is traditionally employed for treating fever, colds, and digestive issues and is recognized for its antiviral, antifungal, and anti-inflammatory properties, particularly against oral mucosa and throat inflammation [41]. In Africa, the empirical medical system also utilizes *M. haplocalyx* for various ailments, including influenza, rheumatism, migraines, ulcers, gastrointestinal disorders, diabetes, psychological and cardiac conditions, and constipation [42]. The potential for developing *M. haplocalyx* in both domestic and international markets is substantial. The diverse therapeutic effects of *M. haplocalyx*, substantiated by traditional applications and potential future uses, warrant further exploration.

## Nutritional value

*M. haplocalyx* is widely recognized for its nutritional components, essential for a quality diet. Its mature and dried stems and leaves are edible [43], and early studies have highlighted its significant role in dietary intake [44]. Proteins, fats, and carbohydrates—key energy sources in human nutrition—are present in substantial amounts in *M. haplocalyx*. Specifically, every 100 g of fresh *M. haplocalyx* contains 6.8 g of protein, 3.9 g of fat, and 67.6 g of carbohydrates (https://www.boohee.com). Additionally, *M. haplocalyx* is rich in amino acids, fatty acids, organic acids, vitamins (B and C), retinol, carotenoids, various nutrients, and trace elements such as sodium, potassium, and phosphorus [45].

Notably, *M. haplocalyx* contains 30% dietary fiber, which is known to benefit metabolic health and reduce the risk of cardiovascular events [46, 47]. Both fresh and dried *M. haplocalyx* also provide relatively high levels of mineral elements, with potassium being the most abundant (135 mg/100 g), followed by iron (4.3 mg/100 g), manganese (5.15 mg/100 g), and zinc (1.64 mg/100 g) (Table 1). These minerals are biologically significant, contributing not only to human metabolic functions and overall health but also supporting the growth, lifecycle, and metabolic functions of plants. Importantly, the minerals in *M. haplocalyx* also serve as excellent dietary antioxidants.

**Table 1:** Nutrition of *M. haplocalyx*

Items	Content (/per 100 g)
Protein	6.8 g
Crude fat	3.9 g
Carbohydrate	67.6 g
Cellulose	31.1 g
K	135 mg
Fe	4.3 mg
Cu	2.08 mg
Mn	5.15 mg
Zn	1.64 mg
P	22 mg
Na	17.5 mg

Proteins, essential for tissue formation and physiological regulation, play a vital role in sustaining human life and health [48]. Amino acids, the building blocks of proteins, form polypeptide chains through peptide bonds [49]. *M. haplocalyx* contains 15 amino acids, including 7 essential ones (threonine, valine, methionine, isoleucine, leucine, phenylalanine, lysine) and 8 non-essential ones (aspartic acid, serine, glutamic acid, glycine, alanine, tyrosine, histidine, arginine) [50]. As a fresh food source, *M. haplocalyx* is rich in both essential and non-essential amino acids, which are pivotal in human physiological processes and significantly contribute to maintaining normal bodily functions and development. Therefore, *M. haplocalyx* can serve as a valuable dietary source of amino acids, supporting the intake of essential amino acids, enhancing bodily functions, and promoting health and quality of life.

*M. haplocalyx* essential oil contains small amounts of fatty acids, including palmitic acid, linoleic acid, and linolenic acid. Although the fatty acid content is low, it still contributes to the nutritional value of *M. haplocalyx*. Moreover, *M. haplocalyx* contains various organic acids, such as caffeic acid, rosmarinic acid, cinnamic acid, citric acid, acetic acid, and malic acid [51]. Rosmarinic acid is the most abundant organic acid, with approximately 156 mg/100 g fresh weight (FW) [52, 53]. These organic acids are important flavor compounds, offering rich taste profiles and being widely used as food flavoring agents in the food industry [54]. The unique taste and aroma of *M. haplocalyx*, attributed to these organic acids, enhance its appeal for both medicinal and culinary applications.

## Phytochemistry

**Fig. 2:**
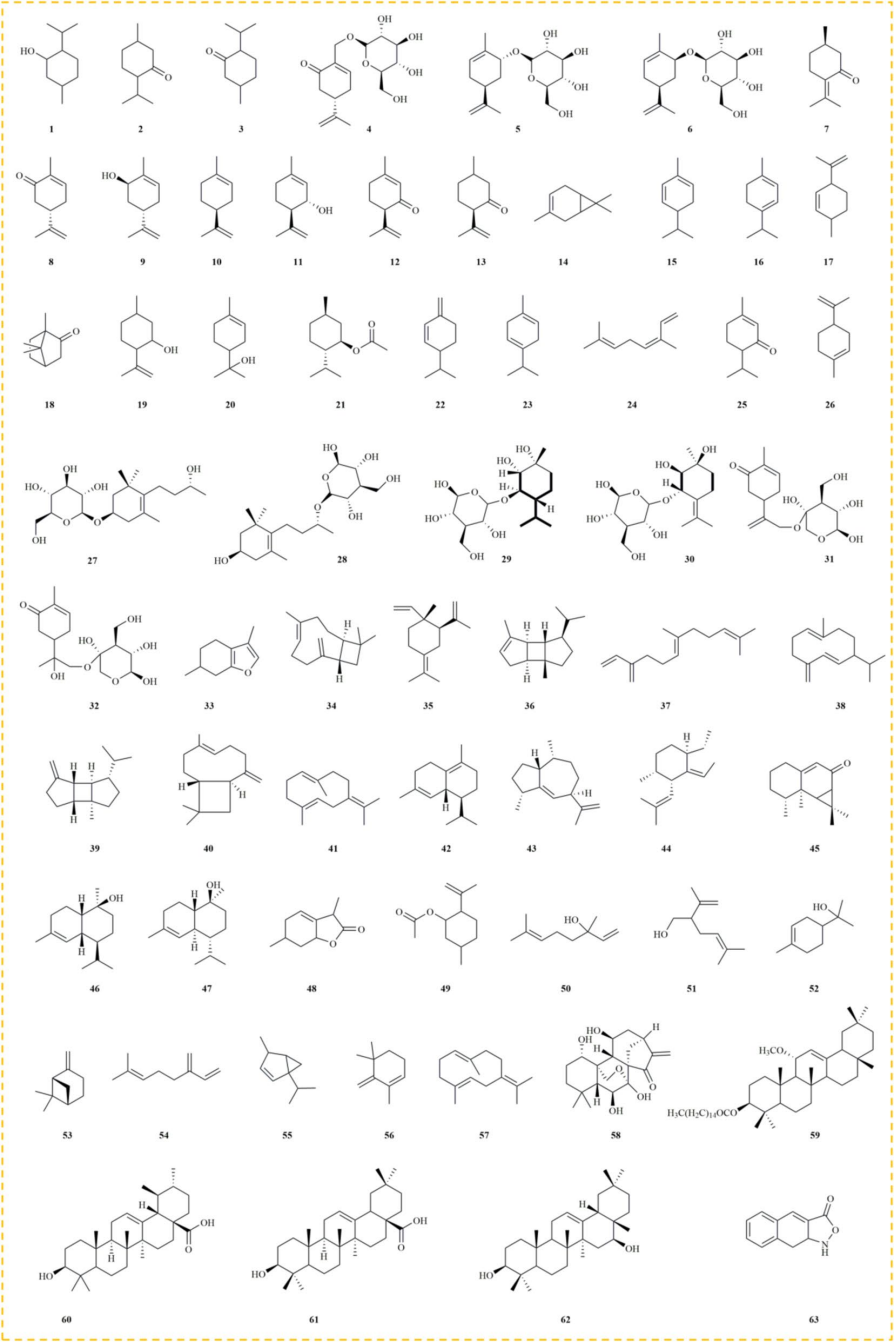
The structures of terpenoids in *M. haplocalyx*

**Fig. 3:**
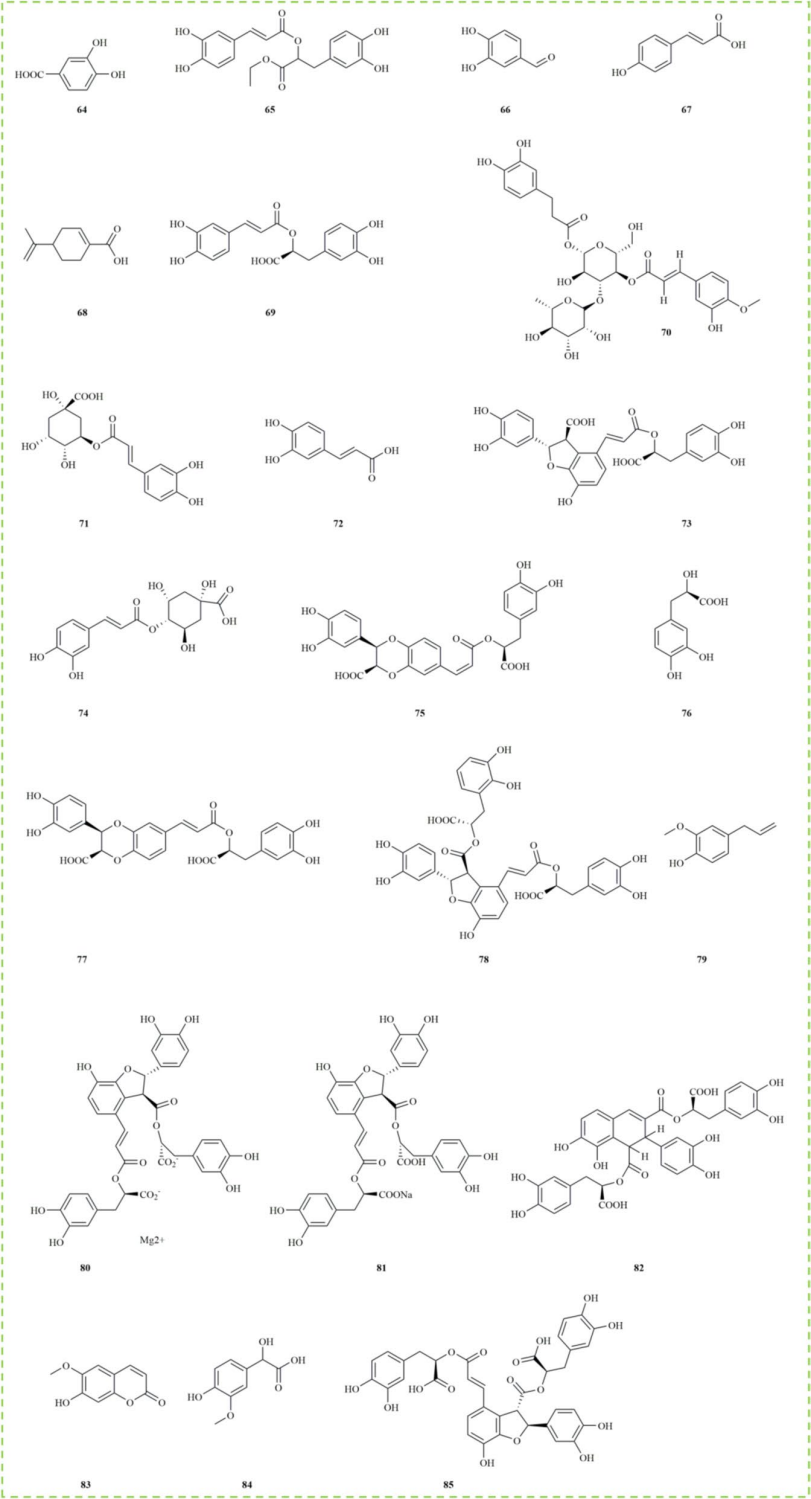
The structures of phenolic acids in *M. haplocalyx*

**Fig. 4:**
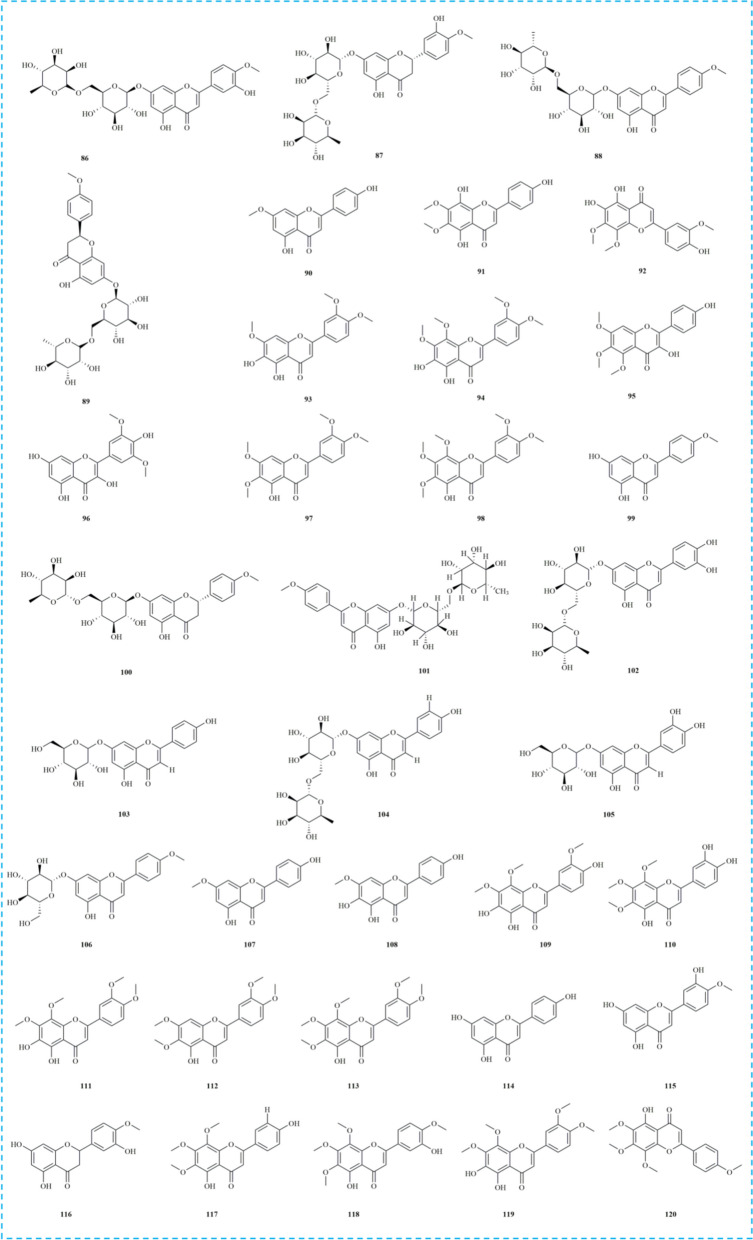
The structures of flavonoids in *M. haplocalyx*

**Fig. 5:**
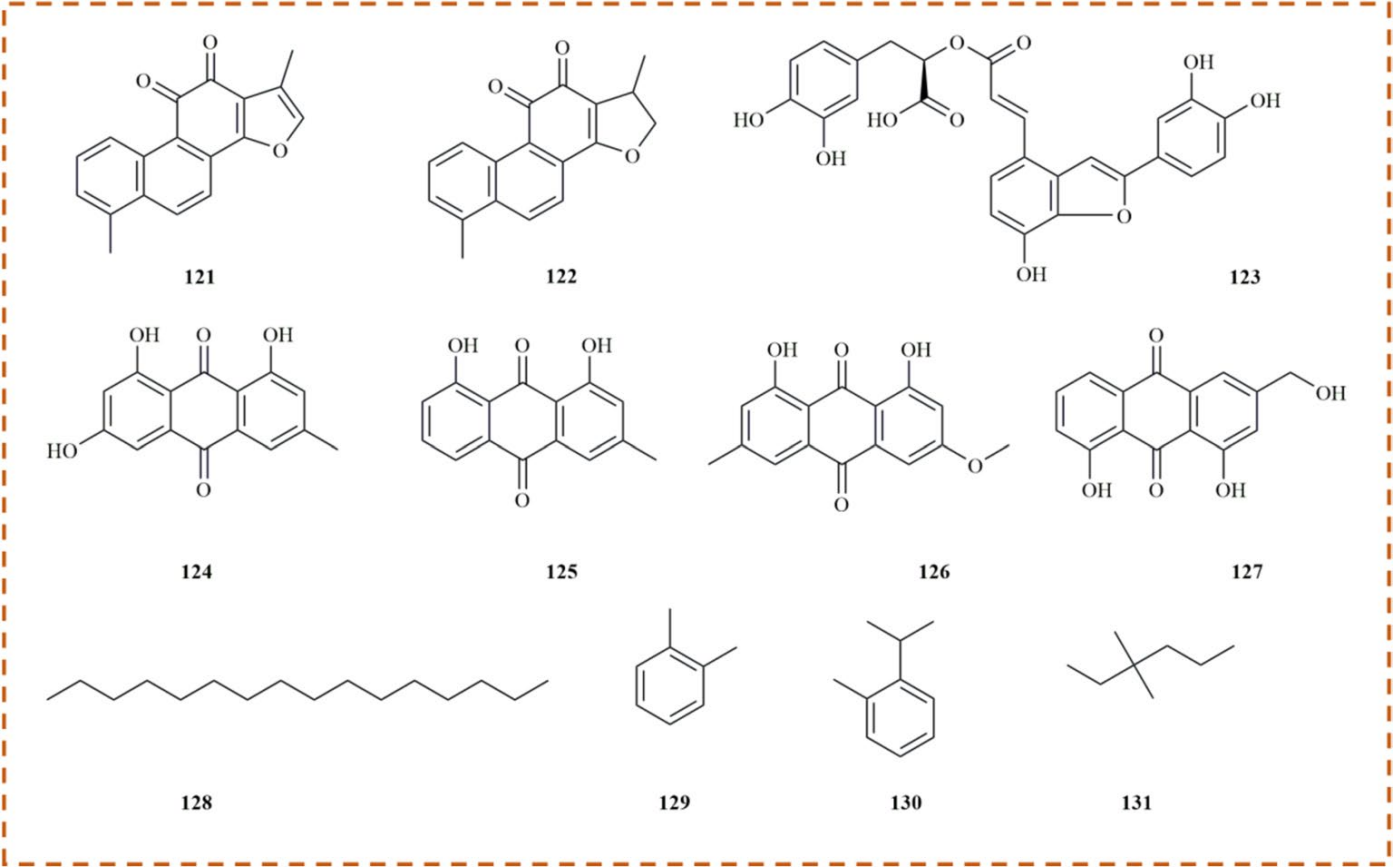
The structures of anthraquinone and alkane in *M. haplocalyx*

**Fig. 6:**
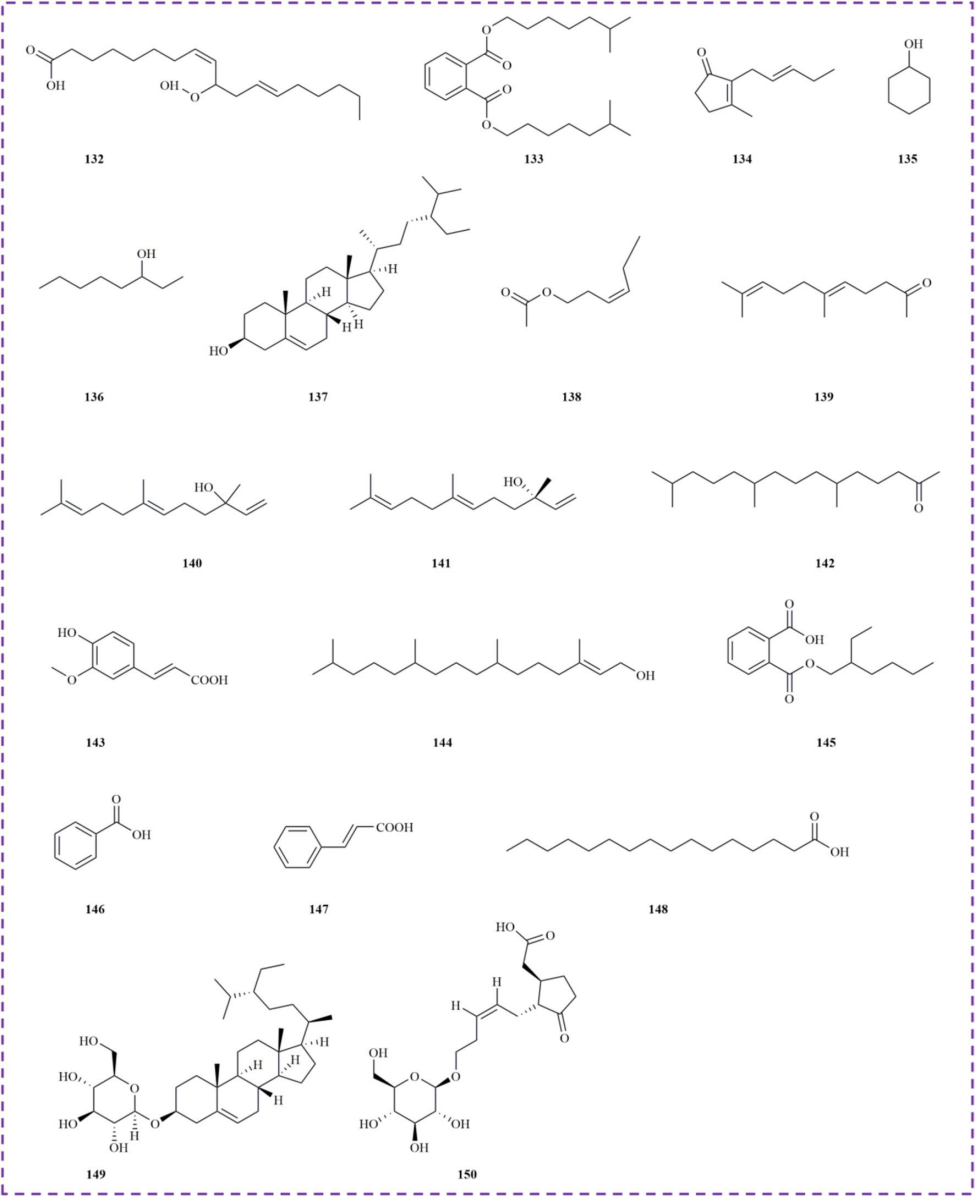
The structures of other compounds in *M. haplocalyx*

Extensive research has identified that *M. haplocalyx* primarily contains terpenoids, flavonoids, phenolic acids, anthraquinones, hydrocarbons, polysaccharides, and other phytochemicals. Among these, volatile compounds like terpenoids are recognized as the primary bioactive constituents, with menthol and menthone being particularly notable. Menthol typically constitutes 62.3–87.2% of *M. haplocalyx*'s volatile components, while menthone accounts for approximately 12% [45]. Notably, menthol serves as the key raw material for *M. haplocalyx* flavoring. These chemical constituents contribute to *M. haplocalyx*'s extensive medicinal properties and nutritional value, making it widely applicable in daily life. Furthermore, the diverse phytochemicals present in *M. haplocalyx* likely play a significant role in its health benefits, as observed in its effects post-consumption. The identified chemical constituents are summarized in Table 2, with their corresponding structures illustrated in Figs. 2, 3, 4, 5, 6.

**Table 2:** Chemical compounds isolated from *M. haplocalyx*

No	Chemical component	Molecular formula	Extraction solvent	Plant parts	References
Terpenoids					
1	Menthol	C_10_H_20_O	Water	Leaves	[73]
2	Isomenthone	C_10_H_8_O	Water	Leaves	[73]
3	Menthone	C_10_H_18_O	Water	Leaves	[73]
4	(4S)-7-hydroxy-carvone 7-*O*-*β*-D-glucopyranoside	C_16_H_24_O_7_	50% ethanol	Aerial parts	[4]
5	(4R,6R)-carveol *β*-D-glucoside	C_16_H_26_O_6_	50% ethanol	Aerial parts	[4]
6	(4R,6S)-carveol *β*-D-glucoside	C_16_H_26_O_6_	50% ethanol	Aerial parts	[4]
7	Pulegone	C_10_H_16_O	Water	Above-ground parts and top leaves	[2]
8	Carvone	C_10_H_14_O	Water	Above-ground parts and top leaves	[2]
9	Trans-carveol	C_10_H_16_O	Water	Above-ground parts and top leaves	[2]
10	Limonene	C_10_H_16_	Water	Above-ground parts and top leaves	[2]
11	Trans-isopiperitenol	C_10_H_16_O	Water	Above-ground parts and top leaves	[2]
12	Isopiperitenone	C_10_H_14_O	Water	Above-ground parts and top leaves	[2]
13	Cis-isopulegone	C_10_H_16_O	Water	Above-ground parts and top leaves	[2]
14	Car-3-ene	C_10_H_16_	Water	Aerial parts	[23]
15	α-Phellandrene	C_10_H_16_	Water	Aerial parts	[23]
16	Terpinene	C_10_H_16_	Water	Aerial parts	[23]
17	Isolimonene	C_10_H_16_	Water	Aerial parts	[23]
18	Camphor	C_10_H_16_O	Water	Aerial parts	[23]
19	Isopulegol	C_10_H_18_O	Water	Aerial parts	[23]
20	α-Terpineol	C_10_H_18_O	Water	Aerial parts	[23]
21	Menthyl acetate	C_12_H_22_O_2_	Water	Aerial parts	[23]
22	β-Phellandrene	C_10_H_16_	Water	Aerial parts	[12]
23	γ-Terpinene	C_10_H_16_	Water	Aerial parts	[12]
24	Cis-ocimene	C_10_H_16_	Water	Aerial parts	[12]
25	Piperitone	C_10_H_16_O	Water	Aerial parts	[12]
26	Cinene	C_10_H_16_	Water	Aerial parts	[73]
27	Linarionoside A	C_19_H_34_O_7_	50% ethanol	Aerial parts	[4]
28	Linarionoside B	C_19_H_34_O_7_	50% ethanol	Aerial parts	[4]
29	Rel-(1R,2S,3R,4R) p-menthane-1,2,3-triol 3-*O*-*β*-D-glucopyranoside	C_16_H_30_O_8_	70% aqueous acetone	Aerial parts	[5]
30	Rel-(1S,2R,3S) terpinolene-1,2,3-triol 3-*O*-*β*-D-glucopyranoside	C_16_H_28_O_8_	70% aqueous acetone	Aerial parts	[5]
31	Spicatoside A	C_16_H_24_O_7_	Water	Whole herbs	[109]
32	Spicatoside B	C_16_H_26_O_8_	Water	Whole herbs	[109]
33	Menthofuran	C_10_H_14_O	Water	Aerial parts	[12]
34	Caryophyllene	C_15_H_24_	Water	Aerial parts	[23]
35	γ-Elemene	C_15_H_24_	Water	Aerial parts	[23]
36	α-Bourbonene	C_15_H_24_	Water	Aerial parts	[23]
37	Farnesene	C_15_H_24_	Water	Aerial parts	[23]
38	Germacrene D	C_15_H_24_	Water	Aerial parts	[23]
39	β-Bourbonene	C_15_H_24_	Water	Aerial parts	[12]
40	β-Caryophyllene	C_15_H_24_	Water	Aerial parts	[12]
41	Germacrene B	C_15_H_24_	Water	Aerial parts	[12]
42	δ-Cadinene	C_15_H_24_	Water	Aerial parts	[12]
43	γ-Gurjunene	C_15_H_24_	Water	Aerial parts	[12]
44	Copaene	C_15_H_24_	Water	Aerial parts	[12]
45	Aristolon	C_15_H_22_O	Water	Aerial parts	[12]
46	Nerolidol	C_15_H_26_O	Water	Aerial parts	[12]
47	Trans-nerolidol	C_15_H_26_O	Water	Aerial parts	[12]
48	Menthalactone	C_10_H_14_O_2_	50% ethanol	Aerial parts	[4]
49	Isopulegol acetate	C_12_H_22_O_2_	Water	Aerial parts	[23]
50	Linaloo	C_10_H_18_O	Water	Aerial parts	[12]
51	Lavandulol	C_10_H_18_O	Water	Aerial parts	[12]
52	Terpineol	C_10_H_18_O	Water	Aerial parts	[12]
53	(1S)-(-)-β-Pinene	C_10_H_16_	Water	Aerial parts	[12]
54	β-Myrcene	C_10_H_16_	Water	Aerial parts	[12]
55	β-Thujene	C_10_H_16_	Water	Aerial parts	[12]
56	1,5,5-trimethyl-6-methylene-cyclohexene	C_10_H_16_	Water	Aerial parts	[12]
57	Cinene	C_10_H_16_	Water	Aerial parts	[12]
58	Lasiodonin	C_20_H_28_O_6_	Water	Leaves	[9]
59	(3β,11α)-3-hydroxy-11α-methoxy-olean-12-en-3-yl palmitate	C_47_H_82_O_3_	50% ethanol	Aerial parts	[4]
60	Ursolic acid	C_30_H_48_O_3_	50% ethanol	Aerial parts	[4]
61	Oleanolic acid	C_30_H_48_O_3_	80% ethanol	Aerial parts	[110]
62	Maniladiol	C_30_H_50_O_2_	50% ethanol	Aerial parts	[4]
63	Naphthisoxazol A	C_11_H_9_NO_2_	50% ethanol	Aerial parts	[4]
Phenolic acids					
64	Protocatechuic acid	C_7_H_6_O_4_	Water	Leaves	[9]
65	Ethyl rosmarinate	C_20_H_20_O_8_	Water	Leaves	[9]
66	Protocatechuic aldehyde	C_7_H_6_O_3_	Water	Leaves	[9]
67	P-coumaric acid	C_9_H_8_O_3_	Water	Leaves	[9]
68	Perillic acid	C_10_H_14_O_2_	Water	Leaves	[9]
69	Rosmarinic acid	C_18_H_16_O_8_	Water	Aerial parts	[3]
70	Eukovoside	C_30_H_38_O_15_	Water	Leaves	[9]
71	Chlorogenic acid	C_16_H_18_O_9_	Water	Leaves	[9]
72	Caffeic acid	C_9_H_8_O_4_	Water	Leaves	[9]
73	Lithospermic acid	C_27_H_22_O_12_	Water	Leaves	[9]
74	Cryptochlorogenic acid	C_16_H_18_O_9_	Water	Leaves	[9]
75	Cis-salvianolic acid J	C_27_H_22_O_12_	70% aqueous acetone	Aerial parts	[8]
76	Danshensu	C_9_H_10_O_5_	70% aqueous acetone	Aerial parts	[8]
77	Salvianolic acid J	C_27_H_22_O_12_	70% aqueous acetone	Aerial parts	[8]
78	Lithospermic acid B	C_36_H_30_O_16_	70% aqueous acetone	Aerial parts	[8]
79	Eugenol	C_10_H_12_O_2_	Water	Aerial parts	[12]
80	Magnesium lithospermate B	C_36_H_28_MgO_16_	70% aqueous acetone	Aerial parts	[8]
81	Sodium lithospermate B	C_36_H_29_NaO_16_	70% aqueous acetone	Aerial parts	[8]
82	Salvianolic acid L	C_36_H_30_O_16_	70% aqueous acetone	Aerial parts	[5]
83	Scopoletin	C_10_H_8_O_4_	Methanol	Aerial parts	[25]
84	Vanillylmandelic acid	C_9_H_10_O_5_	Methanol	Aerial parts	[25]
85	Salvianolic acid B	C_36_H_30_O_16_	Methanol	Aerial parts	[25]
Flavonoids					
86	Diosmin	C_28_H_32_O_15_	Water	Leaves	[9]
87	Hesperidin	C_28_H_34_O_15_	Water	Leaves	[9]
88	Linarin	C_28_H_32_O_14_	Water	Leaves	[9]
89	Isosakuranetin-7-*O*-rutinoside	C_28_H_34_O_14_	Water	Leaves	[9]
90	Genkwanin	C_16_H_12_O_5_	Water	Leaves	[9]
91	Thymusin	C_17_H_14_O_7_	Water	Leaves	[9]
92	Thymonin	C_18_H_16_O_8_	Water	Leaves	[9]
93	5,6-dihydroxy-7,3′,4′-methoxyflavone	C_18_H_16_O_7_	Water	Leaves	[9]
94	5,6-dihydroxy-7,8,3′,4′-tetramethoxy flavone	C_19_H_18_O_8_	Water	Leaves	[9]
95	3,4′-dihydroxy-5,6,7-methoxyflavone	C_18_H_16_O_7_	Water	Leaves	[9]
96	Syringetin	C_17_H_14_O_8_	Water	Leaves	[9]
97	5-hydroxy-6,7,3′,4′-tetramethoxy flavone	C_19_H_18_O_7_	Water	Leaves	[9]
98	5-hydroxy-6,7,8,3′,4′-pentamethoxy flavone	C_20_H_20_O_8_	Water	Leaves	[9]
99	Acacetin	C_16_H_12_O_5_	Water	Leaves	[9]
100	Didymin	C_28_H_34_O_14_	Water	Aerial parts	[3]
101	Buddleoside	C_28_H_32_O_14_	Water	Aerial parts	[3]
102	Luteolin-7-*O*-rutinoside	C_27_H_30_O_15_	Methanol	Aerial parts	[25]
103	Apigenin-7-*O*-glucoside	C_21_H_20_O_10_	Methanol	Aerial parts	[25]
104	Isorhoifolin	C_27_H_30_O_14_	Methanol	Aerial parts	[25]
105	Luteolin-7-*O*-glucoside	C_21_H_20_O_11_	Methanol	Aerial parts	[25]
106	Tilianine	C_22_H_22_O_10_	Methanol	Aerial parts	[25]
107	5,4′-dihydroxy-7-methoxyflavone	C_16_H_12_O_5_	Methanol	Aerial parts	[25]
108	5,6,4′-trihydroxy-7-methoxyflavone	C_16_H_12_O_6_	Methanol	Aerial parts	[25]
109	5,6,4′-trihydroxy-7,8,3′-trimethoxyflavone	C_18_H_16_O_8_	Methanol	Aerial parts	[25]
110	5,3′,4′-trihydroxy-6,7,8-trimethoxyflavone	C_18_H_16_O_8_	Methanol	Aerial parts	[25]
111	5,6-dihydroxy-7,8,3′,4′-tetramethoxyflavone	C_19_H_18_O_8_	Methanol	Aerial parts	[25]
112	5-hydroxy-6,7,3′,4′-tetramethoxyflavone	C_19_H_18_O_7_	Methanol	Aerial parts	[25]
113	5-hydroxy-6,7,8,3′,4′-pentamethoxyflavone	C_20_H_20_O_8_	Methanol	Aerial parts	[25]
114	Apigenin	C_15_H_10_O_5_	Methanol	Aerial parts	[25]
115	Diosmetin	C_16_H_12_O_6_	Methanol	Aerial parts	[25]
116	Hesperetin	C_16_H_14_O_6_	Methanol	Aerial parts	[25]
117	Xanthomicrol	C_18_H_16_O_7_	Methanol	Aerial parts	[25]
118	Gardenin D	C_19_H_18_O_8_	Methanol	Aerial parts	[25]
119	Pebrellin	C_19_H_18_O_8_	Methanol	Aerial parts	[25]
120	Gardenin B	C_19_H_18_O_7_	Methanol	Aerial parts	[25]
Anthraquinone					
121	Tanshinone I	C_18_H_12_O_3_	Water	Leaves	[9]
122	Dihydrotanshinone I	C_18_H_14_O_3_	Water	Leaves	[9]
123	Salvianolic acid C	C_26_H_20_O_10_	Water	Leaves	[9]
124	Emodin	C_15_H_10_O_5_	Water	Aerial parts	[111]
125	Chrysophanic acid	C_15_H_10_O_4_	Water	Aerial parts	[111]
126	Physcion	C_16_H_12_O_5_	Water	Aerial parts	[111]
127	Aloe-emodin	C_15_H_10_O_5_	Water	Aerial parts	[111]
Alkane					
128	Hexadecane	C_16_H_34_	Water	Leaves	[9]
129	*O*-Xylene	C_8_H_10_	Water	Aerial parts	[9]
130	2-isopropyltoluene	C_10_H_14_	Water	Aerial parts	[23]
131	3,3-dimethylhexane	C_8_H_18_	Water	Aerial parts	[23]
Others					
132	Hydroperoxy octadecadienoic acid	C_18_H_32_O_4_	Water	Leaves	[9]
133	Diisooctyl phthalate	C_24_H_38_O_4_	Water	Aerial parts	[23]
134	Jasmone	C_11_H_16_O	Water	Aerial parts	[12]
135	Cyclohexanol	C_6_H_12_O	Water	Aerial parts	[12]
136	3-octanol	C_8_H_18_O	Water	Aerial parts	[12]
137	β-Sitosterol	C_29_H_50_O	80% ethanol	Aerial parts	[110]
138	Cis-3-hexenyl phenyl acetate	C_8_H_14_O_2_	Water	Aerial parts	[12]
139	Geranyl acetone	C_13_H_22_O	Water	Aerial parts	[12]
140	Τ-Muurolol	C_15_H_26_O	Water	Aerial parts	[12]
141	α-Cadinol	C_15_H_26_O	Water	Aerial parts	[12]
142	2-pentadecanone, 6,10,14-trimethyl-	C_18_H_36_O	Water	Aerial parts	[12]
143	Ferulic acid	C_10_H_10_O_4_	Water	Aerial parts	[112]
144	Phytol	C_20_H_40_O	Water	Aerial parts	[12]
145	Mono-ethylhexyl phthalate	C_16_H_22_O_4_	Water	Aerial parts	[12]
146	Benzoic acid	C_7_H_6_O_2_	Water	Aerial parts	[111]
147	Trans-cinnamic acid	C_9_H_8_O_2_	Water	Aerial parts	[111]
148	Palmitic acid	C_16_H_32_O_2_	Water	Aerial parts	[113]
149	Daucosterol	C_35_H_60_O_6_	80% ethanol	Aerial parts	[110]
150	Tuberonic acid glucoside	C_18_H_28_O_9_	Methanol	Aerial parts	[25]

### Terpenoids

Terpenoids, polymers and derivatives of isoprenes, form the backbone of many essential phytochemical components in *M. haplocalyx* [55]. These compounds, characterized by their widespread distribution, complex structures, and significant biological activities, are abundant in *M. haplocalyx*. To date, over 63 terpenoids have been isolated and identified from *M. haplocalyx*, encompassing a broad spectrum of common terpenoids, including monoterpenes, sesquiterpenes, diterpenes, and triterpenes. Specifically, compounds **1–33** and **49–57** are classified as monoterpenes, **34–47** as sesquiterpenes, **58** as a diterpene, and **59–62** as triterpenes [4, 12, 23]. Notably, terpenoids such as menthol **(1)** and menthone **(3)** are the primary bioactive constituents of *M. haplocalyx*, contributing both to its distinctive aroma and its medicinal properties. For instance, menthol has been shown to significantly reduce neuronal cell death, showcasing neuroprotective effects, while menthone exhibits anti-asthmatic potential. An overview of the terpenoids **(1–63)** is presented in Fig. 2.

### Phenolic acids

Phenolic acids, a subclass of plant phenols, are important secondary metabolites widely present in fruits, vegetables, and grains, and are prominently found in *M. haplocalyx* [56]. These compounds are typically conjugated with carbohydrates in the form of glycosides. From a human health perspective, phenolic acids are known to prevent the development of various diseases due to their antioxidant properties [57, 58]. Extensive research indicates that the phenolic acids in *M. haplocalyx* possess diverse biological activities, making them valuable natural phytochemicals with significant research and application potential. Approximately 22 phenolic acids have been isolated from *M. haplocalyx*, including common ones such as protocatechuic acid **(64)**, rosmarinic acid **(69)**, chlorogenic acid **(71)**, and caffeic acid **(72)**. Notably, compounds like lithospermic acid B **(78)**, magnesium lithospermate B **(80)**, and sodium lithospermate B **(81)** were isolated from *M. haplocalyx* for the first time, demonstrating exceptional antioxidant activity in the DPPH radical scavenging assay, with SC50 values of 15.98 μM, 17.85 μM, and 18.22 μM, respectively [8]. Additionally, rosmarinic acid **(69)** exhibited not only strong DPPH scavenging activity but also significantly inhibited ovalbumin (OVA)-induced airway inflammation, suggesting its potential in repairing pathological lung damage caused by inflammation [59]. Furthermore, danshensu **(76)** has been reported to play a critical role in preventing lipid peroxidation and cardiovascular diseases. The structures of these phenolic acids **(64–85)** are depicted in Fig. 3.

### Flavonoids

In recent years, flavonoids among the various phytochemical constituents identified in *M. haplocalyx* have attracted significant attention from researchers due to their unique contributions to the plant's biological properties. Flavonoids are naturally occurring polyphenolic compounds [60], characterized by a 15-carbon skeleton comprising two benzene rings and one heterocyclic ring [61]. Through extensive research, nearly 35 types of flavonoids have been discovered in *M. haplocalyx*, primarily falling into two major subclasses: flavones and flavonols. The flavonoids present in relatively high concentrations in *M. haplocalyx* include hesperidin **(87)**, linarin **(88)**, diosmin **(86)**, and luteolin-7-*O*-glucoside **(105)**. Linarin, in particular, has demonstrated various biological activities in modern pharmacological studies, especially its anti-inflammatory and neuroprotective effects. Additionally, other significant flavonoids in *M. haplocalyx* include acacetin **(99)**, buddleoside **(101)**, tilianine **(106)**, and diosmetin **(115)**. Flavonoids in *M. haplocalyx* not only protect the plant from biotic and abiotic stressors but also contribute to the prevention of neurodegenerative diseases in the human diet [9, 25]. The chemical structures of flavonoids **86–120** are depicted in Fig. 4.

### Anthraquinone

Anthraquinones, another class of natural products with significant biological activities, are commonly found in medicinal herbs and have been extensively studied for their potential applications in various fields [62]. Seven anthraquinone compounds have been isolated and identified from *M. haplocalyx*, including emodin **(124)** and aloe-emodin **(127)**, both of which are derivatives of natural anthraquinones. Emodin is recognized as a protein tyrosine kinase inhibitor and an anti-cancer agent, showing efficacy against various tumor cells. Recent studies have highlighted the diverse health benefits of aloe-emodin from *M. haplocalyx*, garnering global attention (Fig. 5) [63, 64].

### Alkane

Alkanes, consisting solely of carbon and hydrogen atoms, represent one of the simplest types of organic compounds. In *M. haplocalyx*, trace amounts of alkane compounds **(128–131)** have been identified, primarily categorized into cyclic and linear alkanes. These constituents may contribute to the distinctive aromatic characteristics of *M. haplocalyx* (Fig. 5).

### Polysaccharide

Polysaccharides, which are carbohydrate polymers synthesized through dehydration and condensation reactions of multiple monosaccharides, have been increasingly recognized for their significant biological activities and essential roles in various life processes [65, 66]. Extensive research has identified six heteropolysaccharides isolated from *M. haplocalyx*. Notably, Fang et al. (2020) used solvent extraction techniques to isolate four of these polysaccharides, named MHP-W, MHP-C, MHP-A, and MHP-S [67]. Preliminary analysis, including molecular weight (Mw) determination and monosaccharide composition via high-performance gel permeation chromatography (HPGPC), 1-phenyl-3-methyl-5-pyrazolone (PMP) derivatives, and high-performance liquid chromatography (HPLC), revealed that MHP-A had the highest extraction yield at (9.37 ± 0.24) % but the lowest Mw. Conversely, MHP-W exhibited the highest uronic acid content and the largest Mw. Subsequently, over the next two years, the same group identified an additional polysaccharide, PMHP-3, characterized as an acidic polysaccharide with an Mw of 21.82 kDa. PMP derivative analysis and HPLC determined that PMHP-3 consisted of mannose, rhamnose, glucuronic acid, galacturonic acid, glucose, galactose, and arabinose in molar ratios of 1.01:1.82:4.26:19.29:2.46:55.08:16.08, respectively, and had a high purity with a total sugar content of (90.17 ± 1.41) % [68]. Furthermore, Jiang et al. (2020) isolated an antioxidant polysaccharide (WMP) from *M. haplocalyx* with an Mw of 26.91 kDa using water extraction, ethanol precipitation, and gel filtration techniques. Detailed structural analysis through HPLC, methylation analysis, gas chromatography-mass spectrometry (GC–MS), and 1D/2D nuclear magnetic resonance spectroscopy revealed that WMP is a heteropolysaccharide primarily composed of galactose (84.2%), glucose (9.8%), mannose (2.8%), and arabinose (3.2%), featuring a main chain of (1 → 6)-α-D-Galp and (1 → 4,6)-α-D-Galp residues, with a side chain comprising (1 → 6)-α-D-Galp and (1 → 6)-α-D-Glcp residues [69]. Collectively, these polysaccharides from *M. haplocalyx* form the foundation of its notable biological activity, contributing to its extensive health benefits, which continue to attract substantial scientific interest. Table 3 presents a summary of the fundamental characteristics of these polysaccharides.

**Table 3:** Polysaccharides of *M. haplocalyx* plants

No	Name	Extraction solvent	Composition	Molar ratio	Mw (kDa)	Total yield (%)	Activity	References
1	MHP-W	95% ethanol	Man, Rib, Rha, GluA, GalA, Glc, Gal, Ara	3.26:1.06:4.49:1.07:12.34:4.92:43.69:29.18	574.76 kDa (23.03%), 22.70 kDa (28.95%), and 12.09 kDa (48.02%)	6.21	Antioxidant	[67]
2	MHP-C	95% ethanol	Man, Rib, Rha, GluA, GalA, Glc, Gal, Ara	1.76:N/A:5.51:2.89:7.81:4.6.65:46.60:28.78	72.53 kDa (35.06%), 10.88 kDa (13.25%), and 5.84 kDa (51.69%)	7.28	Antioxidant	[67]
3	MHP-A	95% ethanol	Man, Rib, Rha, GluA, GalA, Glc, Gal, Ara	2.51:N/A:3.24:1.92:8.54:7.02:44.44:32.34	11.73 kDa (15.21%) and 6.21 kDa (84.79%)	9.37	Antioxidant	[67]
4	MHP-S	95% ethanol	Man, Rib, Rha, GluA, GalA, Glc, Gal, Ara	1.57:N/A:5.45:2.68:7.80:4.74:44.99:32.76	86.75 kDa (26.01%), 18.27 kDa (48.32%), and 6.29 kDa (25.67%)	7.78	Antioxidant	[67]
5	PMHP-3	Water	Man, Rha, GluA, GalA, Glc, Gal, Ara	1.01:1.82:4.26:19.29:2.46:55.08:16.08	21.82 kDa	12.73	Gut health improvement	[68]
6	WMP	95% ethanol	Gal, Glc, Man, Ara	84.2:9.8:2.8:3.2	26.91 kDa	84.33	Antioxidant and anti-aging	[69]

mannose, (Man); rhamnose, (Rha); ribose, (Rib); galacturonic acid, (GalA); glucuronic acid, (GluA); glucose, (Glc); galactose, (Gal); arabinose, (Ara)

N/A: information was not available

### Others

Beyond these six primary components, an additional 19 compounds **(132–150)** have also been reported from *M. haplocalyx*. Among these, cyclohexanol **(135)**, 3-octanol **(136)**, β-sitosterol **(137)**, and phytol **(144)** are classified as alcohols. Ferulic acid **(143)** and benzoic acid **(146)** are aromatic acids, while trans-cinnamic acid **(147)** and palmitic acid **(148)** are organic acids. Daucosterol **(149)**, a natural sterol identified in *M. haplocalyx*, is notable for its neuroprotective and anti-cancer properties (Fig. 6).

### Biosynthesis

**Fig. 7:**
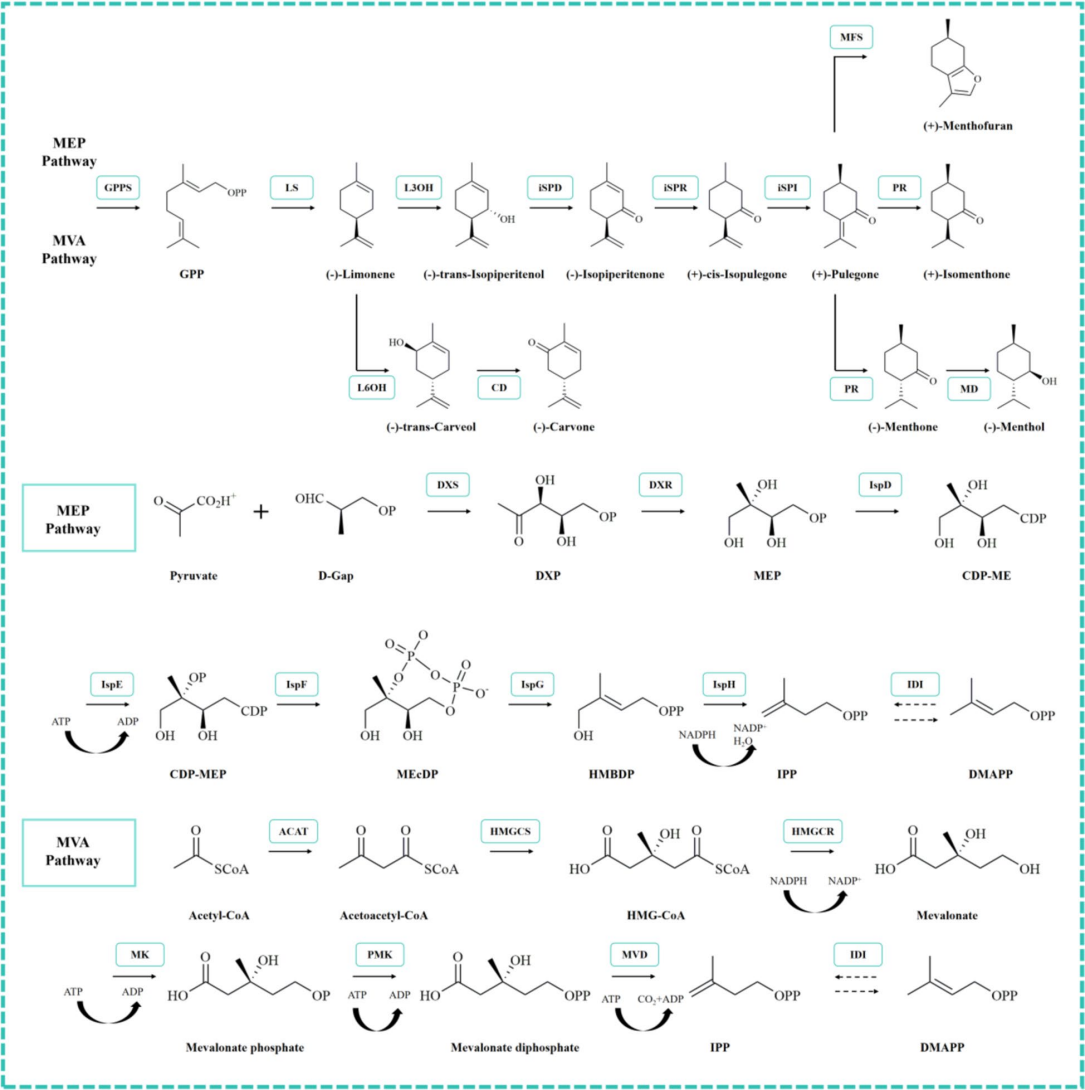
The biosynthesis pathway of main monoterpenes in *M. haplocalyx*. (GPPS, Geranylgeranyl pyrophosphate synthase; GPP, Geranyl diphosphate; LS, (−)-limonene synthase; L6OH, (−)-limonene 6-hydroxylase; CD, (−)-trans-carveol-dehydrogenase; L3OH, (−)-limonene 3-hydroxylase; iSPD, isopiperitenol dehydro-genase; iSPR, (−)-isopiperitenone reductase; iSPI, ( +)-cis-isopulegone isomerase; MFS, ( +)-menthofuran synthase; PR, ( +)-pulegone reductase; MD, (−)-menthol d-ehydrogenase; DXS, 1-Deoxy-D-xylulose 5-phosphate synthase; DXP, 1-Deoxy-D-xylulose 5-phosphate; DXR, 1-deoxy-D-xylulose 5-phosphate reductoisomerase; MEP, 2C-Methyl-D-erythritol 4-phosphate; IspD, 2-C-methyl-D-erythritol 4-phos-phate cytidylyltransferase; CDP-ME, 4-Diphosphocytidyl-2-C-methylerythritol; Is-pE, 4-(cytidine 5′-diphospho)-2-C-methyl-D-erythritol kinase; CDP-MEP, 4-Dip-hosphocytidyl-2-C-methyl-D-erythritol 2-phosphate; IspF, 2-C-methyl-D-erythritol-2,4-cyclodiphosphate synthase; MEcDP, 2C-Methyl-D-erythritol-2,4-cyclodiphosp-hate; IspG, 4-hydroxy-3-methylbut-2-en-1-yl diphosphate synthase; HMBDP, 1-H-ydroxy-2-methyl-2-(E)-butenyl-4-diphosphate; IspH, 4-hydroxy-3-methylbut-2-eny-ldiphosphate reductase; IPP, Isopentenyl diphosphate; IDI, Isopentenyl diphosph-ate isomerase; DMAPP, Dimethylallyl diphosphate; ACAT, Acetyl-coenzyme A acetyltransferases (Thiolase); HMGCS, Hydroxymethylglutaryl-CoA synthase; H-MGCR, 3-Hydroxy-3-methylglutaryl-coenzyme A reductase; MK, Mevalonate kin-ase; PMK, Phosphomevalonate kinase; MVD, Mevalonate diphosphate decarbox-ylase)

*M. haplocalyx* is renowned for its abundant bioactive secondary metabolites, with monoterpenes standing out due to their structural diversity and broad pharmacological properties. Over the past few decades, extensive research has documented the wide array of secondary metabolites present in *M. haplocalyx*, and in recent years, the biosynthesis of these high-value compounds has become a focal point of scientific inquiry. *M. piperita* and *M. haplocalyx*, two widely used species of the *Mentha* genus, play significant roles in the edible and medicinal fields, respectively. Each of these species exhibits distinct characteristics in the biosynthetic pathways of monoterpenes. The biosynthesis of monoterpenes in *M. piperita* and *M. haplocalyx* predominantly occurs via the MEP (methylerythritol phosphate) and MVA (mevalonic acid) pathways [21, 70]. As illustrated in Fig. 7, these pathways involve a sequence of enzyme-mediated reactions, starting with the formation of isopentenyl diphosphate (IPP) and dimethylallyl diphosphate (DMAPP) as precursor molecules. These precursors subsequently combine to generate geranyl diphosphate (GPP), a key intermediate leading to the synthesis of various monoterpenes in *M. haplocalyx*. Notably, the MEP pathway has been identified as the primary route for monoterpene biosynthesis in *M. haplocalyx* [2]. In *M. piperita*, the MVA pathway may also play a crucial role, particularly in the synthesis of its characteristic monoterpenes, such as menthol and menthyl acetate. Moreover, variations in enzyme activity and expression levels between the MEP and MVA pathways can influence the yield and composition of specific monoterpenes in the plant. The biosynthesis of monoterpenoids in *M. haplocalyx* has been extensively studied, and the use of engineering techniques to manipulate their production presents a promising avenue for research [71]. Approaches such as metabolic engineering, genetic engineering, and enzyme engineering hold substantial potential for enhancing monoterpene production in *M. haplocalyx*, particularly given their extensive applications in the pharmaceutical, food, and fragrance industries. Comprehensive investigation and analysis of the monoterpene biosynthetic pathways in *M. haplocalyx* not only facilitate the high-purity production of these commercially valuable compounds but also offer critical insights for future optimization of terpenoid production through advanced engineering methods.

## Health benefits

**Fig. 8:**
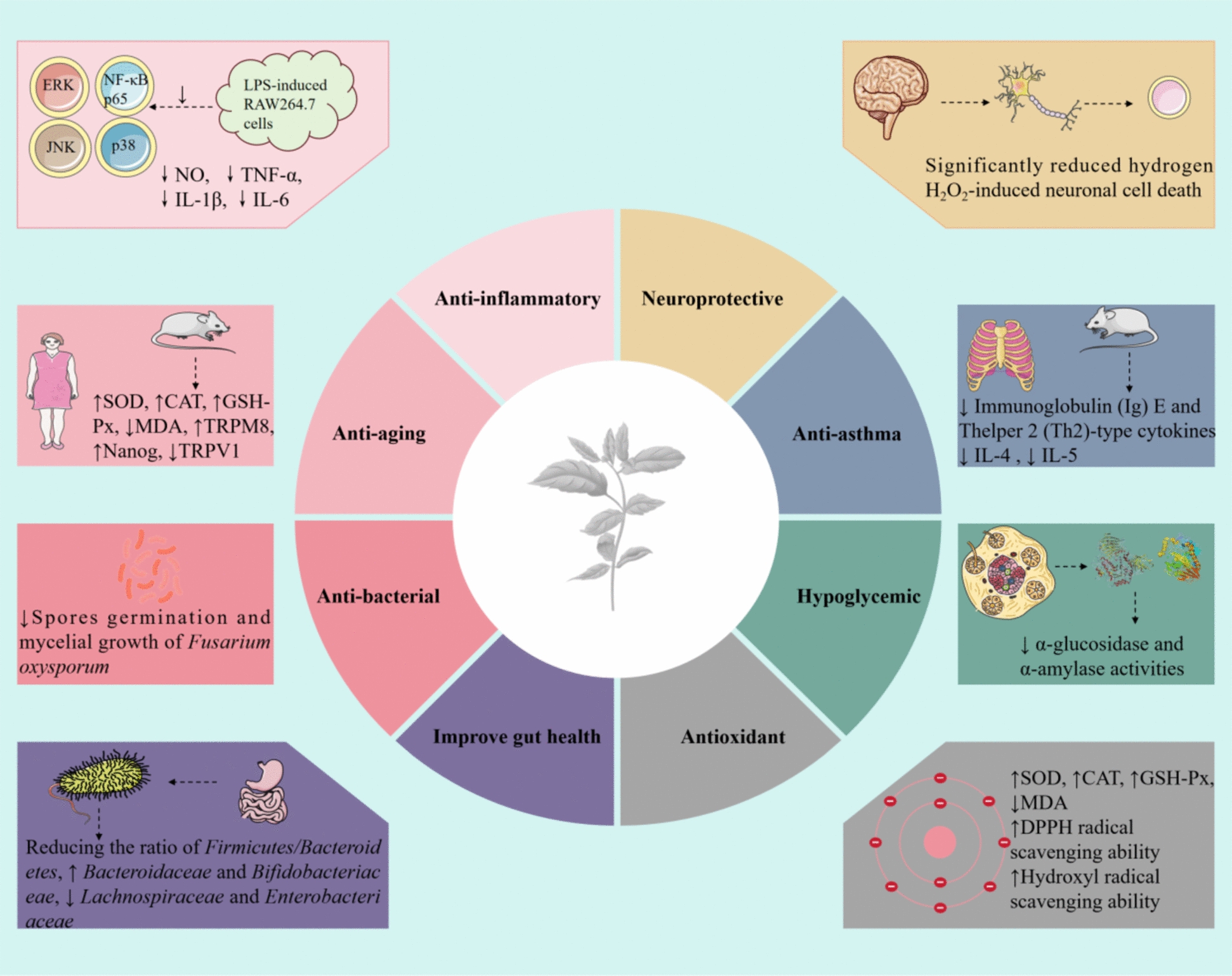
The health benefits of *M. haplocalyx*. (↑): improve or promote. (↓): inhibit or reduce

*M. haplocalyx* is abundant in nutrients and bioactive components, offering a broad spectrum of health benefits, including neuroprotective, anti-asthma, anti-inflammatory, gut health improvement, hypoglycemic, anti-aging, anti-bacterial, and antioxidant activities. Table 4 and Fig. 8 provide an overview of these health benefits.

**Table 4:** Summary of health benefits of *M. haplocalyx* extracts/compounds

Health benefits	Study design	Models	Results/mechanisms	Dosages	References
Neuroprotective	In vitro	H_2_O_2_-induced rat hippocampal neuronal cells	Significantly reduced hydrogen H_2_O_2_-induced neuronal cell death	400 μM	[14]
	In vitro	H_2_O_2_-induced rat hippocampal neuronal cells	The flotation product of menthol could alleviate H_2_O_2_-induced oxidative stress	200 μL	[73]
Anti-asthma	In vivo	Female BALB/c mice (OVA-induced mouse model of allergic asthma)	Significantly inhibited increases in immunoglobulin (Ig) E and T-helper 2 (Th2)-type cytokines such as IL-4 and IL-5 in bronchoalveolar lavage fluid (BALF) and lung tissue	100 mg/kg	[15]
	In vivo	OVA-induced mouse models	Significantly reduced the levels of inflammatory mediators, eosinophil infiltration, and mast cell degranulation in the BALF ↓CC receptor 3 and CXC receptor 1, ↑Th1 cytokine levels	40 mg/kg	[76]
Anti-inflammatory	In vitro	LPS-induced RAW264.7 cells	↓NO, ↓TNF-α, ↓IL-1β, ↓IL-6	50–200 μg/mL 5–20 μM	[79]
Gut health improvement	In vitro	Fresh saliva of healthy volunteers	Reducing the ratio of *Firmicutes/Bacteroidetes*, promoting the proliferation of beneficial bacteria such as *Bacteroidaceae* and *Bifidobacteriaceae*, and inhibiting harmful bacteria such as *Lachnospiraceae* and *Enterobacteriaceae*	Not detected	[68]
Hypoglycemic	In vitro	α-Glucosidase and α-Amylase	The inhibition rates for α-glucosidase and α-amylase activities were (65.34 ± 2.48) % and (45.97 ± 1.13) %	5 mg/mL	[67]
	In vitro	α-Glucosidase	IC50: 21.0 μg/mL	Not detected	[4]
Anti-aging	In vivo	D-Gal-induced mouse model	↑SOD, ↑CAT, ↑GSH-Px, ↓MDA	50 mg/kg	[69]
	In vivo	Mouse and *Caenorhabditis elegans* models	↑TRPM8, ↑Nanog, ↓TRPV1, ↓P53, ↓NF-κB	Not detected	[16]
Anti-bacterial	In vitro	*Fusarium oxysporum*	Inhibition of *Fusarium oxysporum* spore germination and mycelial growth MIC: 0.58 mg/mL	0.5 mg/mL	[51]
Antioxidant	In vivo	D-Gal-induced mouse model	↑SOD, ↑CAT, ↑GSH-Px, ↓MDA	10, 50, and 100 mg/kg	[69]
	In vitro	WMP	↑DPPH radical scavenging ability ↑Hydroxyl radical scavenging ability ↑Ferrous ion chelating activity	1–50 μg/mL 0.5–3.5 mg/mL 1 mg/mL	[69]
	In vitro	MHPs	↑Superoxide radical scavenging activity ↑DPPH radical scavenging activity ↑Hydroxyl radical scavenging activity	0.125, 0.25, 0.5, 1, 2, 3, and 4 mg/mL	[67]
	In vivo	Genetically improved farmed tilapia	↑SOD, ↑CAT, ↑GPx, ↓GSH	0.2, 2.0, 20, and 200 µg/L	[91]

(↑): improve or promote

(↓): inhibit or reduce

### Neuroprotective properties

Neuroprotection involves maintaining nerve tissue function and network integrity while preventing damage caused by pathogens and neurodegenerative diseases. Linarin, a flavonoid glycoside naturally found in *M. haplocalyx*, has garnered attention for its diverse biological effects, particularly in inhibiting the progression of neurodegenerative diseases [72]. Evidence suggests that linarin exerts neuroprotective effects, as demonstrated in a study using H_2_O_2_-induced oxidative stress in rat hippocampal neurons. The oxidative stress model was effectively established by treating cells with 400 μM H_2_O_2_, leading to a marked decrease in cell viability. Neuronal apoptosis was assessed using the DAPI method, revealing that 400 μM H_2_O_2_ significantly increased the number of apoptotic neurons. However, neurons cultured in a medium containing linarin showed a notable reduction in H_2_O_2_-induced neuronal death. These results indicate that linarin in *M. haplocalyx* has a substantial neuroprotective effect, mitigating oxidative stress induced by H_2_O_2_ [14]. Thus, linarin emerges as a promising natural neuroprotective agent, offering valuable insights into potential treatments for nervous system diseases.

Furthermore, research has shown that menthol, another component of *M. haplocalyx*, also exhibits neuroprotective effects against H_2_O_2_-induced oxidative stress in rat hippocampal neurons. A similar oxidative stress model was established with 400 μM H_2_O_2_, which significantly diminished cell viability. The DAPI method revealed a considerable increase in apoptotic neurons under H_2_O_2_ treatment, whereas pre-incubation with menthol significantly reduced neuronal death [73]. These results suggest that menthol in *M. haplocalyx* may possess neuroprotective properties, potentially paving the way for new therapeutic approaches to related diseases. However, it is important to note that most of the current research on the neuroprotective properties of active compounds in *M. haplocalyx* is conducted in vitro, which limits the prospects for future clinical applications. Therefore, further in vivo studies are crucial to validate these neuroprotective effects and explore the safety and efficacy of these compounds in humans.

### Anti-asthma properties

Asthma, a prevalent respiratory disease affecting both children and adults globally, is associated with substantial morbidity, mortality, and economic burden. As a chronic inflammatory immune disorder, asthma is primarily characterized by excessive mucus production in the lungs and inflammatory responses involving various cell types [74, 75]. Recent studies on *M. haplocalyx* have increasingly recognized its anti-asthmatic properties. Lee et al. [15] explored the protective effects of *M. haplocalyx* ethanol extract in an OVA-induced allergic asthma mouse model. Mice were administered *M. haplocalyx* ethanol extract orally at a dose of 100 mg/kg, with montelukast (30 mg/kg) serving as a positive control. The study revealed that *M. haplocalyx* ethanol extract significantly reduced the levels of immunoglobulin (Ig) E and IgG2a in bronchoalveolar lavage fluid (BALF) and lung tissue, as well as the expression of T-helper 2 (Th2)-type cytokines, including IL-4 and IL-5. This inhibition of Th2 cytokines consequently suppressed the infiltration of inflammatory cells into the airways. Additionally, the extract demonstrated antioxidant capacity by reducing reactive oxygen species (ROS) levels in BALF. Histological analysis corroborated these findings, showing reduced eosinophil and macrophage infiltration, alongside decreased mucus cell proliferation and secretion. These results suggest that *M. haplocalyx* ethanol extract may offer therapeutic potential in allergic asthma by modulating immune responses and mitigating oxidative stress [15].

Menthone, a prominent monoterpene in *M. haplocalyx*, also exhibits promising therapeutic effects against allergic asthma. At a dosage of 40 mg/kg, menthone significantly lowered the levels of inflammatory mediators, eosinophil infiltration, and mast cell degranulation in the BALF of an OVA-induced mouse model. Furthermore, it downregulated the gene expression of CC receptor 3 and CXC receptor 1, both closely linked to allergic inflammation, while promoting the restoration of alveolar macrophage proportions. Menthone's ability to regulate the Th1/Th2 immune balance and reduce the ratio of pro-inflammatory to anti-inflammatory cytokines in BALF underscores its potential as an anti-asthmatic agent [76]. Moreover, other constituents of *M. haplocalyx* have demonstrated anti-asthmatic activity. Rosmarinic acid, a natural phenolic compound found in *M. haplocalyx*, has shown significant inhibitory effects on OVA-induced airway inflammation and contributes to the repair of pathological lung damage. Rosmarinic acid exerts its anti-asthmatic effects by significantly reducing mRNA levels of key inflammatory mediators in lung tissue, such as AMCase, CCL11, CCR3, Ym2, and E-selectin. Its therapeutic potential is further enhanced by its regulation of cellular signaling pathways, particularly through the inhibition of extracellular regulated protein kinases (ERK), c-Jun N-terminal kinase (JNK), and p38 mitogen-activated protein kinase (p38 MAPK) phosphorylation, while activating the nuclear factor kappa B (NF-κB) signaling pathway [59]. Taken together, these results highlight the potential of *M. haplocalyx* as a source of new therapeutic strategies for the clinical treatment of allergic asthma and underscore the significant value of this traditional medicinal plant in modern drug development.

### Anti-inflammatory properties

**Fig. 9:**
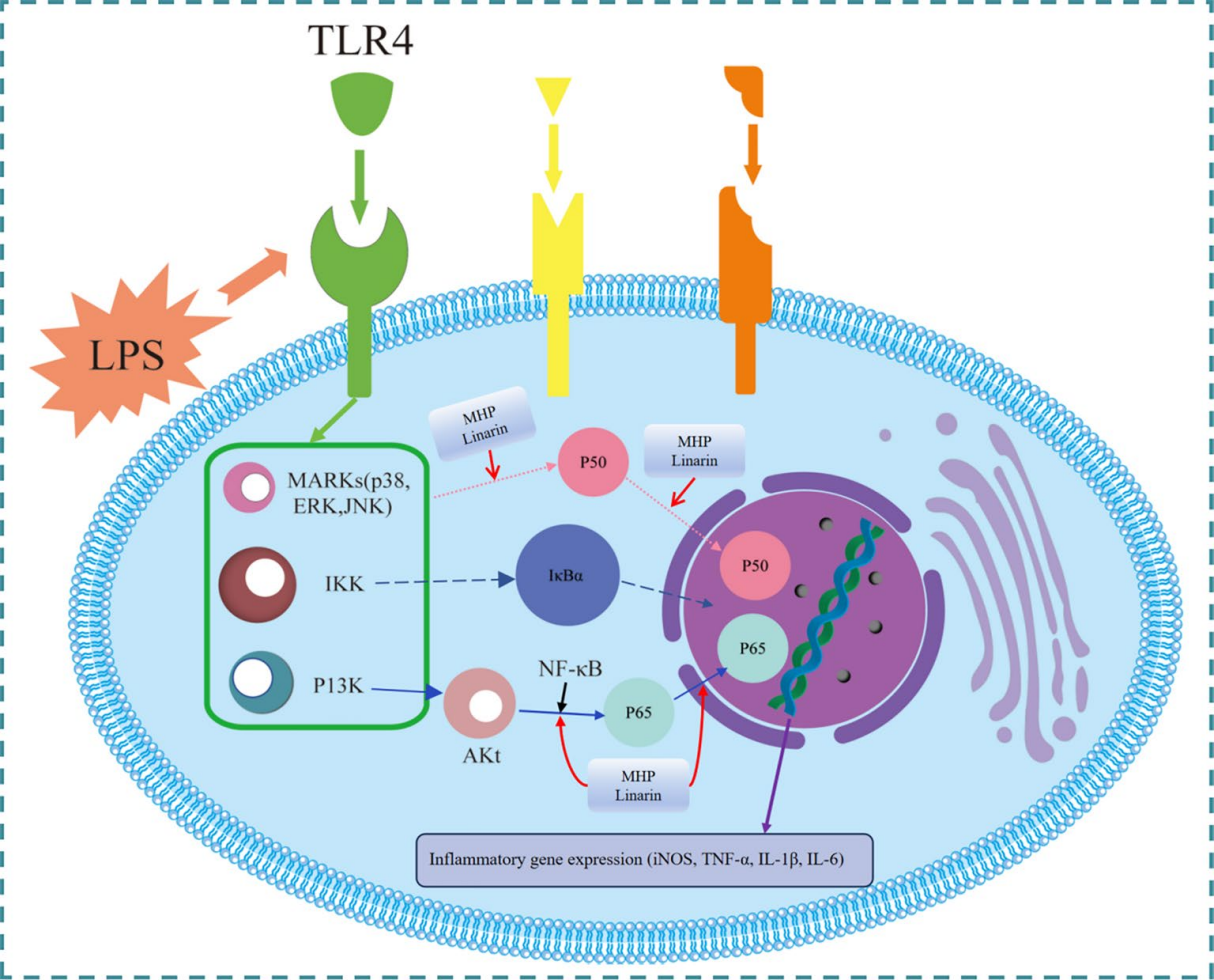
Possible roles of *M. haplocalyx* and linarin in LPS-induced inflammatory responses in RAW264.7 cells. TLR4: Toll-like receptor 4; IKK: IκB kinase; PI3K: phosphoinositide 3-k

Uncontrolled inflammation, triggered by various factors, is one of the most prevalent health issues and can, in severe cases, lead to fatal outcomes. Consequently, the search for effective anti-inflammatory treatments remains a critical focus of medical research [77, 78]. *M. haplocalyx* is recognized for its potent anti-inflammatory properties, which align with its traditional use in heat-clearing and detoxification. Chen et al. [79] demonstrated that the phenolic fraction of *M. haplocalyx*, particularly its active component linarin, exhibits significant inhibitory effects on the production of inflammatory mediators. In a lipopolysaccharide (LPS)-induced RAW264.7 cell model, these compounds, within a dose range of 50–200 µg/mL and 5–20 µM, markedly reduced the levels of nitric oxide (NO), tumor necrosis factor-α (TNF-α), interleukin-1β (IL-1β), and IL-6 in a dose-dependent manner. This reduction was closely associated with the downregulation of inducible nitric oxide synthase (iNOS) mRNA expression. Notably, the anti-inflammatory effects of the phenolic fraction and linarin were comparable to those of dexamethasone, a well-known anti-inflammatory drug. Further mechanistic investigations revealed that the phenolic fraction of *M. haplocalyx* and linarin significantly inhibited the phosphorylation of critical proteins such as p65 and inhibitor kappa B α (IκBα) in the LPS-induced NF-κB signaling pathway, as well as the activation of ERK, JNK, and p38, which are members of the MAPK family. However, these compounds did not significantly affect the phosphorylation of the Akt signaling pathway. These results suggest that the anti-inflammatory effects of the phenolic fraction and linarin are mediated through the inactivation of the NF-κB and MAPK signaling pathways [79]. Figure 9 illustrates the relationship between these effects and the suppression of LPS-triggered NF-κB and MAPKs signaling. This research not only elucidates the molecular mechanisms underlying the anti-inflammatory actions of *M. haplocalyx* but also highlights its potential as a therapeutic agent for the prevention and treatment of inflammatory conditions [80].

### Gut health improvement properties

Disruptions in the balance of the intestinal ecosystem can lead to immune system dysregulation, diminishing the body's disease resistance and potentially triggering various adverse effects, such as metabolic disorders that negatively impact both physical and mental health [81, 82]. Recent research has highlighted the beneficial effects of the polysaccharide PMHP-3, derived from *M. haplocalyx*, on gut health. In an in vitro simulated digestion experiment, PMHP-3 exhibited remarkable stability, as evidenced by the unchanged Mw, total sugar content, and uronic acid content throughout the digestion process, indicating its resistance to digestion. Moreover, in an in vitro fermentation model, PMHP-3 at a concentration of 12.5 mg/mL significantly lowered the pH of the fermentation broth, a change that correlated with enhanced gut microbiota diversity. Specifically, PMHP-3 from *M. haplocalyx* stimulated the growth of beneficial bacteria such as *Bacteroidaceae* and *Bifidobacteriaceae* while inhibiting the proliferation of potentially harmful bacteria like *Lachnospiraceae* and *Enterobacteriaceae*. Additionally, during fermentation, PMHP-3 significantly increased the levels of short-chain fatty acids (SCFAs), including acetic acid, propionic acid, and n-butyric acid, which are essential for maintaining gut health and modulating the host's immune response [68].

In another groundbreaking study, *M. haplocalyx* extract, utilized as a key component in a feed additive, demonstrated positive regulatory effects on the gut microbiota of fattening sheep. The extract notably increased the relative abundance of beneficial bacteria such as *Paraprevotella* and *Alloprevotella*, while reducing the abundance of potentially harmful bacteria like *Blautia* [83]. These results further corroborate the role of *M. haplocalyx* extract in regulating gut microbial balance and promoting the growth of beneficial bacteria. Collectively, these studies provide a strong scientific foundation for the potential application of *M. haplocalyx* in maintaining intestinal function and promoting overall gut health.

### Hypoglycemic properties

Hyperglycemia has been reported to accelerate endothelial cell senescence, thereby contributing to the development of diabetic complications. In critically ill patients, hyperglycemia is also associated with increased mortality. Natural plant polysaccharides, which are high molecular weight substances abundantly found in plants, exhibit a wide array of biological activities [84, 85]. Among these, the polysaccharides derived from *M. haplocalyx* (MHPs) have recently garnered significant attention within the scientific community due to their unique health benefits, particularly their hypoglycemic effects. Further purification of MHPs has yielded four distinct polysaccharides: MHP-W, MHP-C, MHP-S, and MHP-A. In vitro studies assessing the inhibitory activity of these purified polysaccharides on α-glucosidase and α-amylase revealed that different extraction solvents significantly influence their bioactivity. MHP-W, obtained through hot water extraction, demonstrated a (64.42 ± 1.44) % inhibition rate against α-glucosidase and a (44.16 ± 0.96) % inhibition rate against α-amylase at a concentration of 5 mg/mL, underscoring its strong potential as a hypoglycemic agent. Similarly, MHP-A, extracted using 5% NaOH/0.05% NaBH_4_, showed a (60.64 ± 1.01) % inhibition rate against α-glucosidase and a (42.64 ± 1.19) % inhibition rate against α-amylase at the same concentration, displaying a clear dose-dependent response. Although MHP-S, extracted with 0.9% NaCl, exhibited slightly weaker inhibition against α-glucosidase at (56.11 ± 1.52) %, it still achieved a (42.16 ± 1.44) % inhibition rate against α-amylase. Notably, MHP-C, obtained through citric acid extraction, presented the highest inhibition rates at 5 mg/mL, with (65.34 ± 2.48) % against α-glucosidase and (45.97 ± 1.13) % against α-amylase. Although the IC_50_ values of MHP-C were 1.96 mg/mL and 10.14 mg/mL—higher than those of the reference drug acarbose—these findings nonetheless highlight the significant hypoglycemic potential of MHP-C [67].

Additionally, other compounds in *M. haplocalyx* have demonstrated hypoglycemic effects. For instance, *M. haplocalyx* extract has shown significant inhibitory activity against α-glucosidase in vitro, with an IC_50_ value of 21.0 μg/mL, exhibiting a stronger dose-dependent effect compared to acarbose. Further bioactivity-guided separation led to the identification of a key active compound, (3R,9S)-megastigman-5-en-3,9-diol 3-*O*-*β*-D-glucopyranoside, which displayed an IC_50_ value of 83.4 μM, indicating its notable efficacy in inhibiting α-glucosidase [4]. Overall, the active components in *M. haplocalyx* offer valuable insights and serve as key guidance for the development of diabetes treatments.

### Anti-aging properties

Organismal aging is characterized by the gradual decline in cellular function and systemic deterioration across multiple tissues, leading to impaired function and an increased susceptibility to mortality. This physiological aging process is complex, often resulting in diminished enzymatic oxidative capacity, heightened production of free radicals, and the accumulation of their by-products [86, 87]. Research has demonstrated that the plant polysaccharide WMP, derived from *M. haplocalyx*, has the capability to activate the body's antioxidant enzyme systems, thereby delaying the aging process. Jiang et al. [69] highlighted that prolonged administration of D-galactose (D-Gal) induces free radical accumulation and impairs antioxidant enzyme activity, making the D-Gal-induced aging model widely used in aging research and in screening substances with anti-aging properties. In this D-Gal-induced aging model, WMP from *M. haplocalyx* significantly enhanced the activity of antioxidant enzymes in both the serum and liver of mice at doses of 10 mg/kg, 50 mg/kg, and 100 mg/kg. These enzymes include superoxide dismutase (SOD), glutathione peroxidase (GSH-Px), and catalase (CAT). Notably, the 50 mg/kg dose of WMP exhibited superior effects in restoring the antioxidant enzyme system. Compared to the positive control, vitamin C (VC, 100 mg/kg), WMP significantly increased SOD activity by 59.18%, GSH-Px activity by 13.61%, and CAT activity by 26.52%. Additionally, WMP reduced serum malondialdehyde (MDA) levels by 26.81%, further underscoring its significant potential in anti-aging applications [69]. Moreover, *M. haplocalyx* and its active component, menthol, may exert anti-aging effects by modulating members of the transient receptor potential (TRP) channel family, particularly TRPV1 and TRPM8. Through the inhibition of the heat-sensitive TRPV1 channel and activation of the cold-sensitive TRPM8 channel, *M. haplocalyx* and menthol may positively influence healthy longevity. This has been evidenced in models such as mice and *Caenorhabditis elegans*, where TRPM8 activation is associated with extended lifespan. Additionally, studies on rat mesenchymal stem cells (MSCs) have shown that *M. haplocalyx* extract can upregulate the expression of the anti-aging gene Nanog while downregulating aging-related genes such as P53 and NF-κB [16]. These effects likely contribute to enhanced cellular regeneration, reduced oxidative stress, and diminished inflammatory responses. In conclusion, this study provides valuable insights into the potential of *M. haplocalyx* in promoting longevity and extending healthy lifespan.

### Anti-bacterial properties

*M. haplocalyx* is renowned for its potent anti-bacterial properties [88]. Numerous studies have confirmed that extracts and essential oils derived from *M. haplocalyx* exhibit significant anti-bacterial activity against a wide spectrum of both gram-positive and gram-negative bacteria [16]. Of particular interest is the *M. haplocalyx* essential oil nanoemulsion (MNEO), which is prepared using ultrasound with Tween 80 and anhydrous ethanol as stabilizers. MNEO, with an average particle size of just 26.07 nm, presents exceptional promise in antimicrobial applications. Compared to traditional *M. haplocalyx* essential oil solutions, MNEO has shown superior efficacy in inhibiting spore germination and mycelial growth of *Fusarium oxysporum*. This enhanced effectiveness is evident in the minimum inhibitory concentration (MIC) comparison: MNEO's MIC is 0.58 mg/mL, significantly lower than the 3.51 mg/mL required by the traditional essential oil solution, indicating that MNEO can effectively inhibit *Fusarium oxysporum* at much lower concentrations. Further molecular studies have elucidated the mechanisms underlying MNEO's anti-bacterial action. MNEO disrupts critical metabolic pathways and biological processes within *Fusarium oxysporum*, including energy metabolism, meiosis, and ribosome function. Specifically, MNEO markedly reduces the expression of genes involved in glycolysis/gluconeogenesis and starch and sucrose metabolism, leading to a decrease in the accumulation of key metabolites within these pathways, thereby disrupting energy metabolism and arresting fungal growth. Additionally, MNEO impacts genes associated with meiosis and ribosome biogenesis, further inhibiting the reproductive capacity of the fungus [51]. Consequently, *M. haplocalyx* is increasingly recognized as a potential adjunctive treatment for the prevention and management of various infectious diseases.

### Antioxidant properties

Antioxidants play a pivotal role in maintaining human health by reducing the risk of cellular damage caused by free radicals. Free radicals are unstable molecules generated through oxidative reactions that can damage cell membranes, proteins, and DNA, potentially leading to various diseases, including cancer and cardiovascular conditions. Adequate levels of antioxidants are essential for lowering the risk of these illnesses and maintaining normal bodily functions [89, 90]. The antioxidant potential of *M. haplocalyx* extract has been validated through various testing methods [91].

The polysaccharide WMP derived from *M. haplocalyx* has demonstrated notable antioxidant properties. In a DPPH radical scavenging assay, WMP achieved a maximum scavenging rate of (71.49 ± 0.84) % at a concentration of 50 μg/mL, with an IC₅₀ value of 6.21 μg/mL, surpassing the standard set by vitamin C (VC) and indicating superior radical scavenging efficiency. Additionally, in a hydroxyl radical scavenging assay, WMP exhibited excellent dose-dependent scavenging activity across a concentration range of 0.5–3.5 mg/mL, with the scavenging rate increasing significantly from 20% to (86.90 ± 2.56) %. The IC₅₀ value was 1.03 mg/mL, slightly higher than VC's 0.98 mg/mL, further confirming WMP's potent antioxidant capabilities. Moreover, WMP also demonstrated impressive results in the Fe^2^⁺ chelating ability test, achieving 64% chelation efficiency at a concentration of 2 mg/mL, with an IC₅₀ value of 1.58 mg/mL. This suggests WMP's strong potential in preventing iron-induced oxidative stress [69].

Similarly, the four polysaccharides (MHPs) from *M. haplocalyx*—MHP-A, MHP-C, MHP-S, and MHP-W—not only exhibit hypoglycemic effects but also significant antioxidant properties. Among these, MHP-C stood out in the DPPH radical scavenging assay, achieving the highest scavenging rate of (79.31 ± 0.70) % at a concentration of 4 mg/mL, with an IC₅₀ value of 1.16 mg/mL, indicating its superior efficacy compared to other polysaccharides. MHP-C also demonstrated the strongest performance in the reducing power test, nearly matching the reducing capacity of ascorbic acid. In contrast, although MHP-A had the highest extraction yield and showed strong superoxide anion radical scavenging ability, its performance in the DPPH radical scavenging assay was less prominent, likely due to its lower molecular weight and higher protein and total phenolic content, which may be more effective in scavenging superoxide anion radicals rather than DPPH radicals. Collectively, these results highlight the potent antioxidant properties of *M. haplocalyx* polysaccharides, establishing them as powerful natural antioxidants [67]. Furthermore, the crude acetone–water extract of *M. haplocalyx* has been validated for its antioxidant activity in the DPPH radical scavenging assay, with an IC₅₀ value of 45.67 μg/mL. Chemical analysis revealed that the phenolic acid compounds isolated from this extract effectively neutralize DPPH radicals through their abundant phenolic hydroxyl groups, thereby inhibiting radical-mediated oxidative stress [8]. It is recommended to further investigate the safety and reliability of the phenolic acids in *M. haplocalyx* for their potential development as antioxidant drugs.

## Application and commercialization potential of *M. haplocalyx* products

### Application of *M. haplocalyx* products

*M. haplocalyx* holds significant medicinal and nutritional value and exhibits broad potential across various industries. Currently, there are 572 patents related to *M. haplocalyx* worldwide (https://www.lens.org/), primarily focused on functional foods, medicine, cosmetics, and other applications. In the food industry, the distinctive aroma and health-promoting properties of *M. haplocalyx* leaves have garnered considerable attention. As a key ingredient in food flavoring, *M. haplocalyx* leaves are used not only in fresh foods but also as a raw material for producing volatile extracts, which are then incorporated into a wide range of beverages and confectioneries [3, 31, 92]. Additionally, *M. haplocalyx* is used in desserts, biscuits, chocolates, and ice cream, imparting a refreshing taste and aroma. Moreover, by combining *M. haplocalyx* with other ingredients such as *Fagopyrum tataricum*, *Poria cocos*, *Pueraria lobata*, and *Glycyrrhiza uralensis*, and processing them through soaking, extraction, filtration, filling, and sterilization, a health tea can be produced. Guided by TCM principles, this tea is believed to aid digestion and provide soothing effects, making it particularly suitable for individuals with hypertension, hyperlipidemia, and hyperglycemia, thus presenting promising industrial prospects. Furthermore, the terpenes and their oxidized derivatives in *M. haplocalyx* essential oil exhibit significant inhibitory effects on mold toxin formation [93], suggesting its potential as a flavoring agent to inhibit the growth of *Aspergillus flavus* and the production of *aflatoxin* in food.

In the pharmaceutical industry, *M. haplocalyx* is increasingly valued for its therapeutic potential. A study revealed that an herbal combination of *M. haplocalyx*, *Coptis chinensis*, *Paeonia lactiflora*, and *Ligusticum chuanxiong* effectively improves or prevents symptoms related to headaches [94]. Notably, extracts and essential oils of *M. haplocalyx* possess analgesic, sedative, and anti-bacterial properties, making them common ingredients in pharmaceutical formulations such as mouth lozenges, oral solutions, and ointments. For instance, mouth lozenges containing *M. haplocalyx* are highly effective in treating and preventing oral ulcers [95]. Additionally, *M. haplocalyx* ointment can be used to some extent in treating mild skin burns; its active ingredients offer anti-itch and analgesic effects, helping to alleviate discomfort in the affected area [96]. The cooling sensation provided by *M. haplocalyx* also helps reduce inflammation and swelling, offering relief to the skin. Furthermore, *M. haplocalyx* essential oil has shown remarkable benefits in aromatherapy. When used in methods such as aromatherapy lamps and massage oils, it can invigorate the mind and soothe muscles [97, 98]. As holistic health continues to gain emphasis, the market demand for *M. haplocalyx* in aromatherapy is likely to grow, further expanding its industrial applications.

**Fig. 10:**
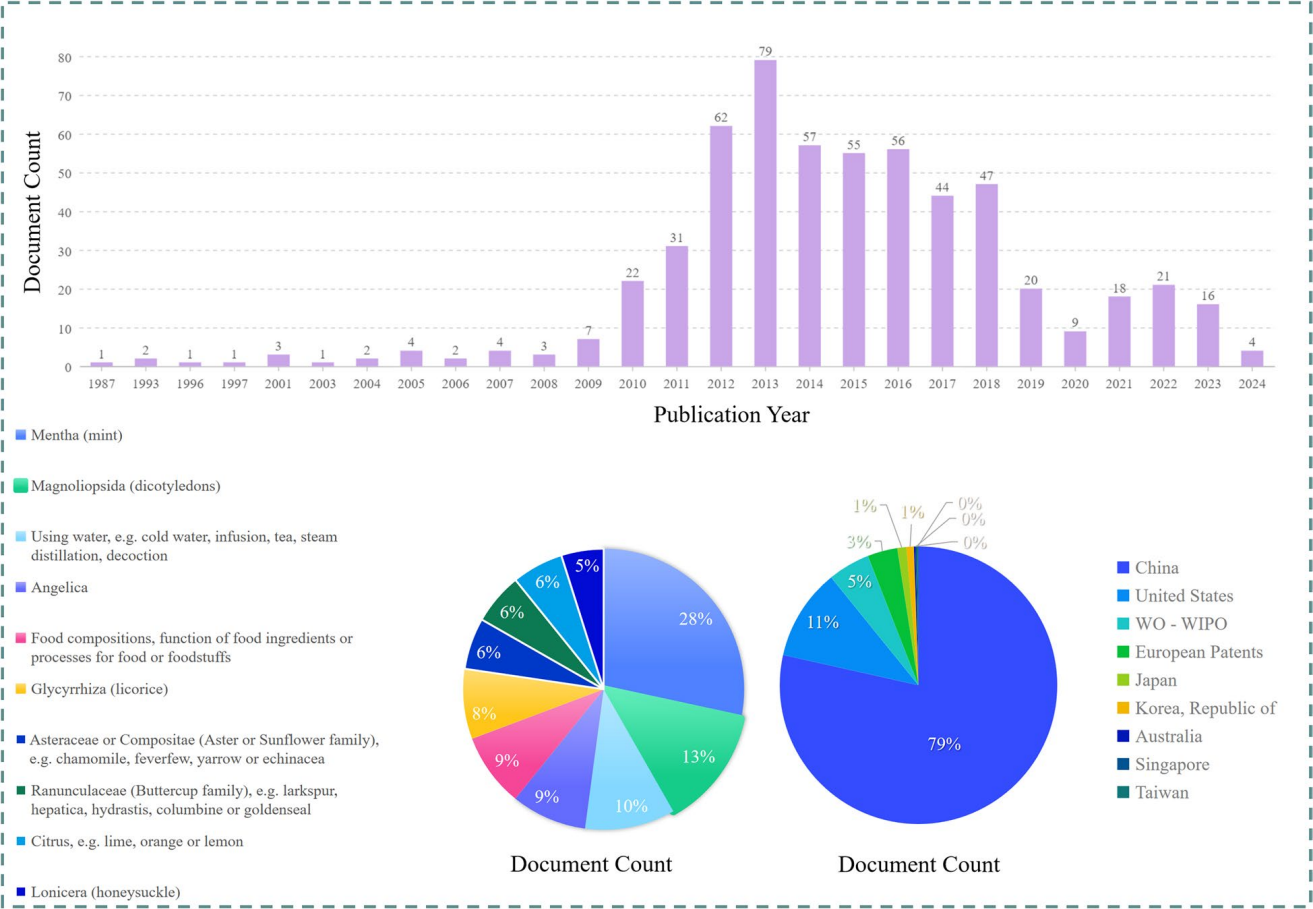
Current situation of patent inventions related to *M. haplocalyx*. **A** Document numbers, **B** Application of patents, **C** Patent distribution

**Fig. 11:**
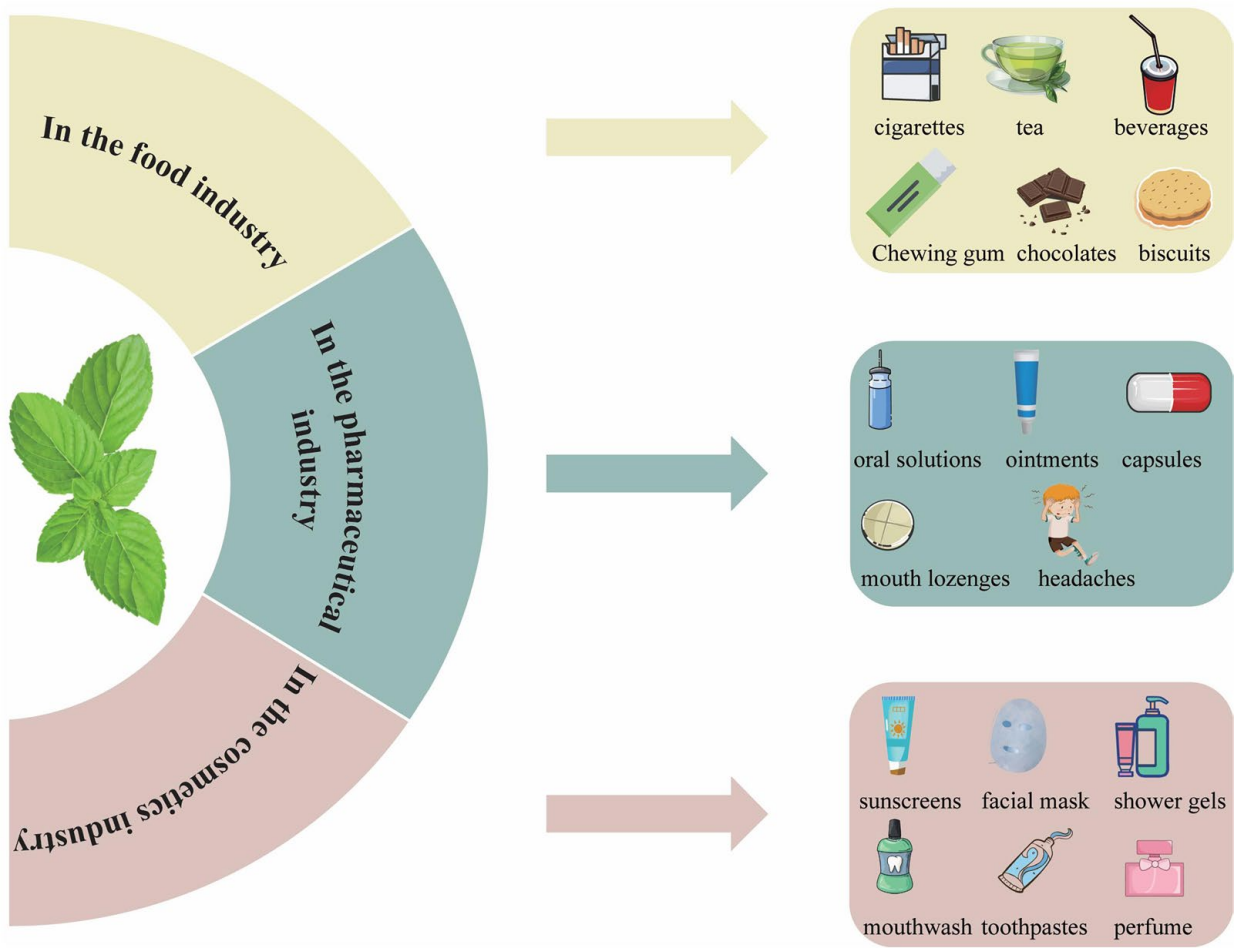
Practical and potential applications of *M. haplocalyx*

In the cosmetics industry, *M. haplocalyx* has gained considerable popularity due to its potent antioxidant properties and the tyrosinase inhibition activity of its essential oil. This essential oil effectively enhances the efficacy of sunscreens, largely attributed to its high sun protection factor (SPF) value [99]. Studies suggest that blemish creams formulated with *M. haplocalyx* extract and *Pueraria lobata* extract exhibit notable whitening effects. Specifically, *M. haplocalyx* extract comprises 45–99.9% of the total mass of tyrosinase inhibitors in these formulations, while *Pueraria lobata* extract makes up 0.1–55%. This formulation, due to its gentle yet effective inhibition of tyrosinase activity, provides a remarkable whitening effect. Additionally, *M. haplocalyx* extract is a common ingredient in oral care products such as toothpaste and mouthwash, where it contributes to fresh breath and oral hygiene [100]. The cool and refreshing fragrance of *M. haplocalyx* is also leveraged in perfume blending, enhancing the scent's freshness. Furthermore, *M. haplocalyx* essential oil is frequently incorporated into skincare products such as facial cleansers, shampoos, and shower gels. In conclusion, with the ongoing research into the unique nutritional and cosmetic properties of *M. haplocalyx*, its potential for further development in the cosmetics industry is significant. In the field of animal husbandry, the application of *M. haplocalyx* as an animal feed additive is gaining attention [101, 102]. There is an increasing trend of incorporating *M. haplocalyx* extracts into animal feed to combat microbial infections and inflammation, particularly in addressing post-weaning diarrhea in livestock and poultry. More importantly, *M. haplocalyx* extracts have been shown to significantly improve gut health and enhance growth performance in animals by modulating gut microbiota and boosting antioxidant capacity [83]. This positions *M. haplocalyx* extracts as a promising natural feed additive for future animal husbandry, particularly in promoting sustainable and healthy breeding practices. As illustrated in Fig. 10, the current status of related patented inventions of *M. haplocalyx* in recent years reveals that China and the United States dominate the patent landscape, accounting for 79% and 11% of the patents, respectively. In contrast, the World Intellectual Property Organization (WO-WIPO) and Europe represent a smaller portion of the patents, with 5% and 3% respectively. Overall, research and development related to *M. haplocalyx* products are still in their early stages. Nonetheless, *M. haplocalyx* is already widely used across diverse fields, including functional foods, medicine, cosmetics, and animal husbandry, as depicted in Fig. 11.

### Commercialization potential of *M. haplocalyx* products

As previously noted, *M. haplocalyx*, a multifunctional medicinal plant with a long history of cultivation in China, has now been widely introduced to many countries, including the United Kingdom, the United States, Japan, South Korea, India, and South Africa [103]. Initially cultivated for ornamental purposes, *M. haplocalyx* was not considered a commercial medicinal plant. However, in recent years, its pleasant aroma, refreshing flavor, and nutritional value have elevated it to the status of a commercial crop [104]. Consequently, the global export value of *M. haplocalyx* has seen continuous growth. With its widespread application in diverse fields such as food, medicine, cosmetics, and agriculture, global demand for *M. haplocalyx* products is on the rise. Many countries that produce *M. haplocalyx* have further stimulated export growth by enhancing cultivation, extraction, and processing technologies while expanding their international markets. These products include *M. haplocalyx* essential oil, tea, chewing gum, and oral care items. As a versatile and nutritious medicinal plant, *M. haplocalyx* appeals to health-conscious consumers. Countries like Mexico, Indonesia, Nigeria, and Turkey have substantial import and export scales for *M. haplocalyx*, reflecting the strong demand in their domestic and foreign markets [30, 105]. *M. haplocalyx* is a commercially viable crop that is easy to cultivate, maintain, and care for. It is rarely affected by diseases or pests, making it a popular secondary crop often grown in orchards and alongside other local crops [106, 107]. As the demand for fresh *M. haplocalyx* products and essential oils continues to rise both domestically and internationally, the cultivation area of *M. haplocalyx* has expanded accordingly. Notably, *M. haplocalyx* cultivation is cost-effective and can be grown as a secondary crop alongside other plants, making it ideal for small-scale farmers in areas with low land occupancy. Cultivating *M. haplocalyx* offers significant economic and social benefits, aligning with the principles of the bio-circular-green (BCG) economic model. As a natural, renewable medicinal plant, *M. haplocalyx* cultivation not only avoids the depletion of natural resources but also promotes sustainable production through proper management and cultivation techniques. The plant's strong photosynthetic capabilities also contribute to mitigating the impacts of climate change. Furthermore, the applications of *M. haplocalyx* extracts and essential oils in various industries have been proven to meet green product standards, thereby contributing to environmental preservation and supporting local economic development by creating jobs and income for farmers and communities [108]. In summary, the cultivation of *M. haplocalyx* presents numerous advantages, including low costs, high yields, and strong market demand. Its potential in both domestic and international markets holds significant practical value for increasing farmers' income and promoting regional economic development.

## Conclusion and prospects

In recent years, *M. haplocalyx* has garnered significant attention from nutritionists, food researchers, and natural plant and herbal research institutions, leading to a thorough exploration and application of its potential value. *M. haplocalyx* is abundant in various nutrients, including essential and non-essential amino acids, organic acids, fatty acids, vitamins, trace elements, high-quality dietary fiber, and structurally diverse phytochemicals. To date, 150 constituents have been successfully isolated from *M. haplocalyx*, with terpenoids, phenolic acids, and flavonoids being the most prominent. These phytochemical compounds contribute to the wide range of health benefits associated with *M. haplocalyx*, such as neuroprotective, anti-asthmatic, anti-inflammatory, hypoglycemic, gut health improvement, and anti-bacterial properties. This article provides the first comprehensive review of *M. haplocalyx*, encompassing its botanical morphology, traditional uses, nutritional value, phytochemistry, health benefits, and practical applications across the food, cosmetics, and pharmaceutical industries.

Despite the significant progress in *M. haplocalyx* research, several issues remain unresolved. Firstly, the abundance of *Mentha* species has led to frequent misidentification and mixing of plants within the genus. Accurately identifying the species of *M. haplocalyx* is a pressing issue that requires resolution. Enhancing the clinical efficacy of *M. haplocalyx* necessitates distinguishing between different species based on plant morphology, component types and concentrations, pharmacological activities, and genetic characteristics. Furthermore, the current Ch.P 2020 uses menthol content (no less than 0.20%) as the sole indicator for assessing the quality of *M. haplocalyx*. However, relying on a single component is insufficient to fully reflect the plant's quality and does not align with the holistic principles of TCM. Therefore, establishing new detection methods or bridging the gaps in quality control from a biological activity perspective is essential.

Secondly, the identification of chemical constituents in *M. haplocalyx* remains incomplete. While recent studies have focused primarily on isolating and identifying terpenoids and volatile essential oils, emerging pharmacological research highlights the potential value of non-volatile components in treating respiratory, reproductive, and digestive system diseases. The development and application of advanced separation techniques are essential for comprehensively identifying and purifying the active compounds in *M. haplocalyx*. This will not only substantiate its potential in clinical treatments but also provide a robust scientific foundation for future drug development. Additionally, it is important to establish systematic methods to evaluate the synergistic effects of multiple active components in *M. haplocalyx* to achieve multi-target drug therapy strategies.

Thirdly, while recent studies have highlighted the neuroprotective and anti-inflammatory potential of *M. haplocalyx*, most of this research has been conducted in vitro or using animal models. The mechanisms of action are not well understood, and no clinical trials of *M. haplocalyx* extracts have been reported to date. Thus, further investigation into the active components of *M. haplocalyx* and their mechanisms of action is necessary to advance its therapeutic applications and translate these findings into clinical practice.

Fourthly, given the diversity of high-value compounds in *M. haplocalyx* and the complexity of its interactions with biological systems, the current research on the plant's potential toxicity is insufficient. Assessing the safety of *M. haplocalyx* is particularly critical, not only because of its widespread use in clinical treatments but also due to its common application in functional foods, cosmetics, and animal feed additives. Therefore, it is imperative to thoroughly investigate any potential adverse effects, determine the safe dosage range, and elucidate the mechanisms underlying its toxic effects to ensure its safety across various applications.

In conclusion, the reviewed literature provides compelling evidence that *M. haplocalyx* possesses significant nutritional value and functional characteristics. There is an urgent need for further research on *M. haplocalyx* in both in vitro and in vivo settings, and even in clinical practice, to develop new therapeutic modalities for various diseases. This paper offers a systematic and comprehensive review of *M. haplocalyx*, aiming to establish a foundation for further investigation into its mechanisms of action and future applications. Additionally, we hope this review will uncover new and intriguing insights about *M. haplocalyx* and provide valuable guidance for the continued development of this natural medicinal plant.

## Data Availability

Not applicable.

## Abbreviations

BALF: Bronchoalveolar lavage fluid
Ch.P 2020: 2020 Edition of the Pharmacopoeia of the People’s Republic of China
DAPI: 4′,6-Diamidino-2-Phenylindole
D-Gal: D-galactose
ERK: Extracellular signal-regulated kinase
FOS: Fructo-oligosaccharides
GSH-Px: Glutathione peroxidase
HPLC: High-performance liquid chromatography
iNOS: Induced nitric oxide synthase
IPP: Isopentenyl diphosphate
JNK: C-Jun NH2-terminal kinase
Mw: Molecular weights
MEP: Methylerythritol phosphate
MSCs: Mesenchymal stem cells
MIC: Minimum inhibitory concentration
OVA: Ovalbumin
qRT-PCR: Quantitative real-time polymerase chain reaction
SOD: Superoxide dismutase
SCFAs: Short-chain fatty acids
TNF-α: Tumor necrosis factor-α
VC: Vitamin C
BCG: Bio-circular-green
CAT: Catalase
DCF: Dichlorofluorescein
DMAPP: Dimethylallyl diphosphate
FW: Fresh weight
GC-MS: Gas chromatography-mass spectrometry
GPP: Generate geranyl diphosphate
HPGPC: High-performance gel permeation chromatography
IL-1β: Interlenkin-1β
IκBα: Inhibitor kappa B α
LPS: Lipopolysaccharide
MDA: Malondialdehyde
MVA: Mevalonic acid
MNEO: *M. haplocalyx* essential oil nanoemulsion
NF-κB: Nuclear factor kappa B
PMP: 1-phenyl-3-methyl-5-pyrazolone
ROS: Reactive oxygen species
SPF: Sun protection factor
TCM: Traditional Chinese medicine
TRP: Transient receptor potential
XYS: Xiaoyaosan
